# Synonymous Mutations in *rpsT* Lead to Ribosomal Assembly Defects That Can Be Compensated by Mutations in *fis* and *rpoA*

**DOI:** 10.3389/fmicb.2020.00340

**Published:** 2020-03-06

**Authors:** Anna Knöppel, Dan I. Andersson, Joakim Näsvall

**Affiliations:** Department of Medical Biochemistry and Microbiology, Uppsala University, Uppsala, Sweden

**Keywords:** synonymous mutations, r-protein S20, ribosome assembly, *fis*, *rpoA*

## Abstract

We previously described how four deleterious synonymous mutations in the *Salmonella enterica rpsT* gene (encoding ribosomal protein S20) result in low S20 levels that can be compensated by mutations that restore [S20]. Here, we have further studied the cause for the deleterious effects of S20 deficiency and found that the S20 mutants were also deficient in four other 30S proteins (S1, S2, S12, and S21), which is likely due to an assembly defect of the S20 deficient 30S subunits. We examined the compensatory effect by six additional mutations affecting the global regulator Fis and the C-terminal domain of the α subunit of RNA polymerase (encoded by *rpoA*). The *fis* and *rpoA* mutations restored the S20 levels, concomitantly restoring the assembly defect and the levels of S1, S2, S12, and S21. These results illustrate the complexity of compensatory evolution and how the negative effects of deleterious mutations can be suppressed by a multitude of mechanisms. Additionally, we found that the mutations in *fis* and *rpoA* caused reduced expression of other ribosomal components. Notably, some of the *fis* mutations and the *rpoA* mutation corrected the fitness of the *rpsT* mutants to wild-type levels, although expression of other ribosomal components was reduced compared to wild-type. This finding raises new questions regarding the relation between translation capacity and growth rate.

## Introduction

The bacterial ribosome consists of three rRNA molecules and ∼50 ribosomal proteins (r-proteins). Maturation of the rRNAs and assembly to the r-proteins to produce the two ribosomal subunits occur in a step-wise process that require several maturation and assembly factors (Held and Nomura, 1973; Kaczanowska and Rydén-Aulin, 2007; Shajani et al., 2011; Sashital et al., 2014). Synthesis of rRNA is controlled by global regulators, such as Fis, DksA, and (p)ppGpp, whereas many of the r-proteins are primarily regulated at a post-transcriptional level through the availability of naked rRNA (Bokal et al., 1995, 1997; Bénard et al., 1996; Hirvonen et al., 2001; Zhang et al., 2002; Murray et al., 2003; Schlax and Worhunsky, 2003; Merianos et al., 2004; Paul et al., 2004). If the available binding sites on the rRNA are saturated by r-protein, the surplus of the protein bind to their own mRNAs to repress translation initiation, effectively balancing r-protein synthesis to rRNA transcription.

Ribosomal protein S20 (encoded by the *rpsT* gene) has been subject to in-depth studies of post-transcriptional auto-regulation and mRNA decay (Parsons and Mackie, 1983; Mackie, 1987, 1991; Parsons et al., 1988; Rapaport and Mackie, 1994; Spickler et al., 2001; Luciano et al., 2012). S20 regulates its own synthesis, most likely by binding to stem-loop structures that overlap the translation initiation codon in its own mRNA, although *in vitro* experiments have failed to demonstrate direct binding (Donly and Mackie, 1988; Burgos et al., 2017). 30S subunits lacking S20 are defective in translation initiation and docking of the two ribosomal subunits (Götz et al., 1990; Rydén-Aulin et al., 1993; Tobin et al., 2010). Previously, we characterized mutations that compensated for the fitness costs of four synonymous *rpsT* mutations in *Salmonella enterica* (Knöppel et al., 2016). The deleterious effects of the synonymous mutations were caused by reduced expression of S20, which presumably lead to accumulation of a sub-population of 30S particles lacking S20. The growth defect could be ameliorated by intragenic suppressor mutations in *rpsT*, copy number variants of the *rpsT* gene, and mutations in the RNA polymerase sigma factor σ70 which all lead to increased expression of the S20 protein.

Apart from these suppressor mutations, several evolved lineages contained mutations in the nucleoid-associated global transcription regulator Fis or in the C-terminal domain of the α subunit of RNA polymerase (αCTD). As mutations in both *rpoA* and *fis* have been found to be beneficial under various growth conditions in different organisms and strain backgrounds, we initially thought these mutations were general adaptations to the laboratory growth conditions (Charusanti et al., 2010; Crozat et al., 2010, 2011; Maharjan et al., 2012; Le Gac et al., 2013). However, in an unrelated study where we identified and characterized a large number of mutations in both *S. enterica* and *Escherichia coli* that conferred adaptation to four common laboratory media, we did not find any mutations affecting αCTD or *fis* in *S. enterica* under any condition (Knöppel et al., 2018). In light of this, we re-assessed the role of these mutations as direct compensations for the synonymous *rpsT* mutations. Both αCTD and Fis are, in addition to their roles in global transcription regulation, key players in the regulation of ribosomal RNA transcription during exponential growth (Estrem et al., 1998; Hirvonen et al., 2001; Maeda et al., 2015), making it plausible that the mutations could compensate for the deleterious *rpsT* mutations in a specific way. The high transcription of rRNA during rapid growth is largely due to binding of the αCTD to UP-elements upstream of the *rrn* P1 promoters, and this is further enhanced by binding of Fis. The absence of UP-elements or Fis sites results in dramatic reductions in transcriptional output (20–50 fold, or 3–8 fold, respectively; Ross et al., 1990, 1998; Bokal et al., 1995; Estrem et al., 1998; Hirvonen et al., 2001). Part of the action of Fis on *rrn* promoters is due to a direct interaction between αCTD and Fis, and the affected amino acid in our *rpoA* mutants is adjacent to a known interaction site (Bokal et al., 1995, 1997; Aiyar et al., 2002; McLeod et al., 2002).

In this study, we further defined the effects of S20 deficiency and found support for an assembly defect of the 30S subunit, potentially resulting in accumulation of dysfunctional 30S subunits lacking S20 and four other proteins. We also characterized the effects of the adaptive mutations in *rpoA* and *fis*. Interestingly, they did not restore S20 expression, but rather reduced the transcription of rRNA. Thus, these findings suggest that the loss of fitness caused by the skewed stoichiometry between S20 and rRNA can be restored in two ways: (i) by restoring S20 expression (Knöppel et al., 2016) and (ii) by lowering the rRNA expression to match the low S20 levels (this study). In both cases, the skewed S20 to ribosome ratio in the synonymous mutants is corrected, relieving the apparent assembly defect. As ribosomes lacking S20 are defective in several steps in translation initiation, reducing the pool of dysfunctional 30S subunits by matching the rate of ribosome synthesis with the low rate of S20 synthesis would allow for more efficient translation initiation and increased fitness.

## Results and Discussion

### S20 Deficiency Lead to Lower Levels of Four Additional r-Proteins, Suggesting an Assembly Defect of the 30S Subunit

**FIGURE 1:**
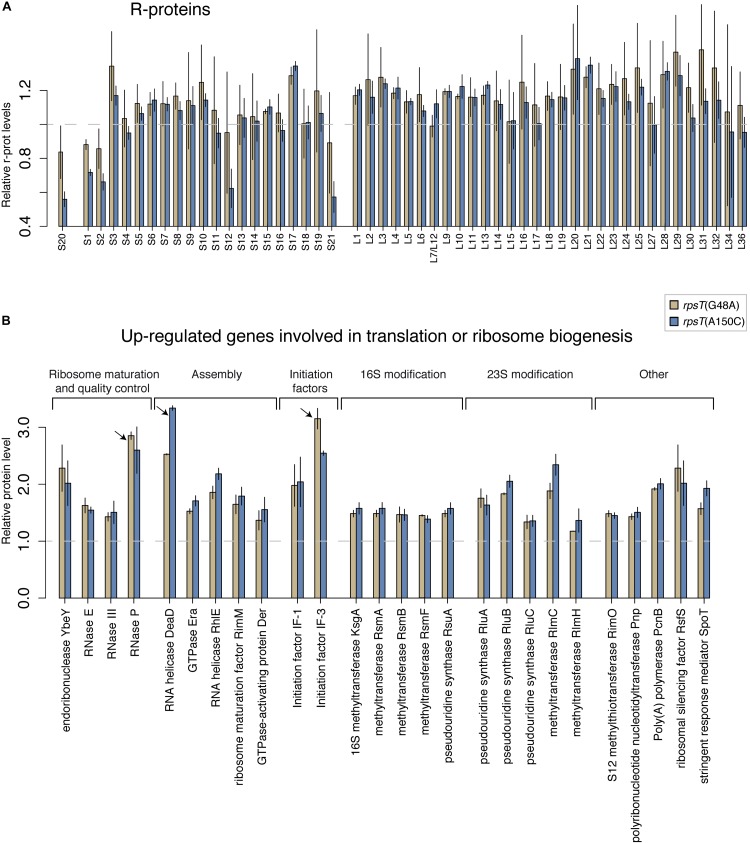
Relative protein levels in the S20 deficient *rpsT* mutants G48A (red) and A150C (turquoise). **(A)** Quantification of relative r-protein levels in G48A and A150C *rpsT* mutants through LC-MS/MS. The reported values are normalized to the average levels of the same protein in the wild-type control (*rpsT*^+^) strain (dashed line) and represent the average of two (G48A) or four (A150C) replicates. **(B)** Ribosome-associated genes that were found to be upregulated. This functional group of genes was overrepresented among the upregulated genes (Supplementary Table S1). Proteins that were the second and third most overexpressed among all genes are marked with arrows.

**FIGURE 2:**
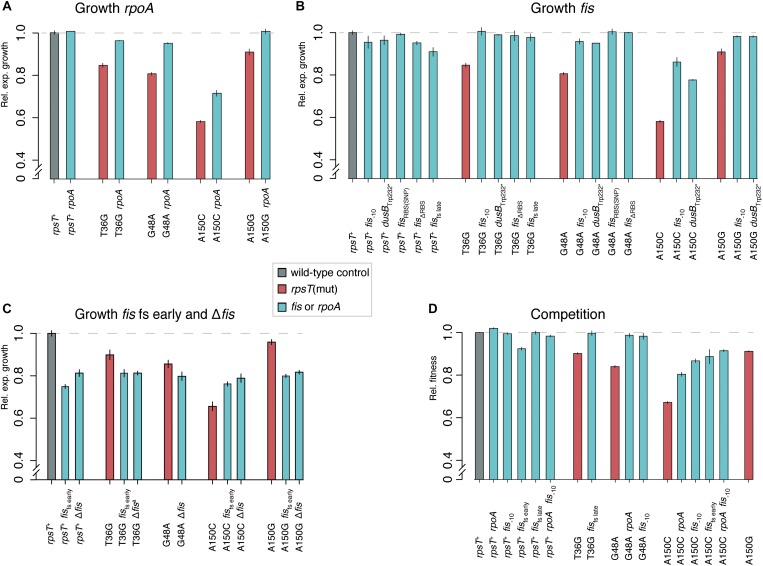
Fitness measurements of *rpoA* and *fis* mutants with and without *rpsT* mutations. All fitness measurements are relative to isogenic wild-type controls (set at 1.0). Reported values represent the mean (±SD) of at least four independent experiments for growth rate measurements and six for competitions. Gray = isogenic wild-type controls; red = *rpsT* mutants, turquoise = *rpoA* or *fis* mutants in wild-type or *rpsT* mutant background. **(A–C)** Relative exponential growth rate of re-constituted or constructed mutants. **(D)** Relative fitness in competition.

In a previous study, we described how four fitness-reducing synonymous mutations in *rpsT* cause low levels of the S20 protein by reducing the steady state levels and translation of the *rpsT* transcript (Supplementary Figure S1; Knöppel et al., 2016). These mutations were T36G (∼90% of wt fitness, ∼60% of normal S20 levels), G48A (∼85% fitness, ∼84% S20), A150C (∼67% fitness, ∼55% S20), and A150G (∼91% fitness, ∼75% S20). To better understand the pleiotropic effects caused by S20 deficiency, we re-analyzed the relative levels of all ribosomal proteins in the two most severe synonymous mutants (A150C and G48A) through LC-MS/MS (Figure 1A). In agreement with our previous study, the S20 levels were lower compared to the bulk of r-proteins and correlated with the fitness of the synonymous mutants (Figure 2A). Interestingly, we additionally found that the 30S r-proteins S1, S2, S12, and S21 were reduced to similar levels as S20. The effect correlated with the growth defects and was clearly seen for the most severe mutant (A150C), whereas the effect was less evident for G48A.

To examine if S20 deficiency caused specific downregulation of these four proteins, we generated *yfp* translational fusions to *rpsL* (S12; which was reduced in the *rpsT* mutants) and *rpsF* (S6; which was not reduced in the *rpsT* mutants), and measured their expression in exponentially growing cells with or without the synonymous *rpsT* mutations present (Supplementary Figure S2). If the *rpsT* mutations reduced the expression (transcription or translation) of the four proteins, we would expect a decrease in YFP signal from the *rpsL* translational fusion relative to the *rpsF* translational fusion in an isogenic strain. However, we found that S20 deficiency reduced YFP expression from both of these fusions to similar relative levels, arguing against any specific effect of S20 deficiency on expression of S12. Instead, this finding suggests that the four proteins S1, S2, S12, and S21 are expressed to similar levels as other ribosomal proteins, but a substantial fraction are subsequently lost without becoming part of 30S subunits. Thus, our results are consistent with a 30S assembly defect, where insufficient levels of the primary binder S20 causes a reduction in S1, S2, S12, and S21 levels by impairing their incorporation into 30S subunits during ribosome assembly. The results also indicate that any surplus of these four proteins that does not end up in mature 30S subunits is degraded and not seen in the proteomics analysis. We also observed that many genes associated with ribosome biogenesis and RNA processing were overrepresented among the most upregulated proteins in the S20 deficient mutants (Figure 1B and Supplementary Table S1). A plausible explanation for this observation is that the cells sense a reduced translation capacity and try to compensate by upregulating these factors.

Proteins S2, S12, and S21 are tertiary binders in the assembly of the 30S subunits and depend on the binding of both primary (e.g., S20) and secondary binders (Held and Nomura, 1973; Moll et al., 2002; Culver, 2003; Talkington et al., 2005; Shajani et al., 2011). Binding of S1 is dependent on the binding of S2 (Moll et al., 2002). Resembling our results, the well-studied 21S ribosome assembly intermediate in most studies lacks or has low levels of the affected r-proteins S1, S2, S12, and S21 (Held and Nomura, 1973; Kaberdina et al., 2009).

Similar to our S20 deficient mutants, treatment with the antibiotic kasugamycin leads to accumulation of 61S ribosomes, with misassembled small subunits lacking r-proteins S1, S2, S6, S12, S18, and S21 (Kaberdina et al., 2009). Unexpectedly, these kasugamycin-induced ribosomes are functional *in vitro* but are restricted to translation from leaderless transcripts. Along the same line, a temperature-sensitive *rpsB* (S2) mutant accumulate small ribosomal subunits lacking S1 and S2 at the non-permissive temperature, leading to a severe reduction in translation from leader-containing mRNAs (Kaberdina et al., 2009). The loss of translation from leader-containing mRNAs has been suggested to result from the lack of the RNA chaperone activity of S1, which is needed to melt RNA structures around the Shine-Dalgarno sequence (Moll et al., 2002; Kaberdina et al., 2009). Accordingly, a mutant that has two populations of 30S subunits (a major, fully assembled population and a minor, misassembled population lacking S1 and other proteins) would have lower overall capacity to translate, due to having fewer fully assembled ribosomes, but would not lose as much capacity in translating leaderless transcripts since the misassembled ribosomes can translate them. Thus, proteins from leaderless transcripts would appear to be over-expressed because most other proteins are under-expressed. We analyzed our *rpsT* mutants with respect to the relative levels of the products from known leaderless transcripts in *S. enterica*. Out of the 14 genes known to have leaderless mRNA (Kröger et al., 2012) and that were found in our LC-MS/MS dataset, seven were more abundant in the *rpsT* mutants than in the wild-type (Supplementary Figure S3). Thus, it is likely that the *rpsT* mutants accumulate a subpopulation of aberrant 30S subunits that due to the lack of S1 behave similarly to subunits that accumulate upon kasugamycin treatment and in S2 mutants.

### S20 Deficiency of Synonymous Mutants Cannot Be Overcome by Auto-Regulation

As expression of S20 is auto-regulated at the level of translation initiation, the wild-type is expected to produce a small surplus of S20 protein, and this free S20 protein binds its own mRNA to stop further translation. Mutants that make less S20 per 16S rRNA are expected to have very low levels of free S20 protein. Consequently, the auto-regulation in such mutants should be de-repressed. To further test the effects of S20 deficiency we analyzed the impact of low S20 levels on S20 autoregulation. To this end, we inserted an *rpsT*^*wt*^ translational *yfp* fusion (Knöppel et al., 2016) into a neutral position on the *Salmonella* genome in strains with the synonymous *rpsT* mutations and compared *yfp* expression to a strain with the wild-type *rpsT* allele (Supplementary Figure S4). The S20-YFP fusion peptide produced from this gene fusion only contains part of S20, and does not complement an S20-deficient mutant. Thus, expression of S20-YFP in S20-deficient mutants reflect effects on *rpsT* autoregulation. As expected for the S20 deficient mutants, an increase in expression of the S20-YFP fusion was observed. The effects correlated negatively with the fitness of the mutants (*R*^2^ = 0.75). These results indicate that the reduced expression of S20 from the native locus in the S20-deficient mutants resulted in lower levels of free S20 protein, which caused weaker auto-repression of S20 synthesis. Apparently, expression from the wild-type *rpsT* gene is high enough to cause significant amounts of auto-repression, while expression from the synonymous mutants studied here is so inefficient that not even de-repression of S20 autoregulation can compensate the low levels. Alternatively, the mutants tested here are all affected in the mechanism of auto-regulation, such that S20 expression from the defective (synonymously mutated) mRNA cannot be sufficiently de-repressed.

### Experimental Evolution to Compensate for S20 Deficiency

In a previous study, we evolved the four deleterious synonymous *rpsT* mutants in a rich growth medium for 200 generations to select for compensatory mutations in an attempt to elucidate potential explanations for the fitness cost of the *rpsT* mutations (Knöppel et al., 2016). Our previous study focused on suppressor mutations that, in different ways, restored the low S20 levels and increased fitness. Here we focused on examining the fitness restoring effects of mutations in *rpoA* and in the *dusB*-*fis* operon, which were present in the same evolved strains.

The mutations in *rpoA* and *dusB*/*fis* are listed in Table 1 and all mutations found in the 24 whole-genome sequenced and evolved *rpsT* mutants, as well as isogenic wild-type control lineages evolved in parallel, are listed in Supplementary Table S2. In *rpoA*, the mutation Leu270Phe in the αCTD domain was found in two independently evolved *rpsT* mutants carrying the synonymous mutations G48A and A150G, respectively. For the *dusB*/*fis*-operon, six different mutations in the evolved strains with the *rpsT* mutations T36G, G48A, and A150C were found. Fis is a global transcription regulator and the mutations in the *dusB*/*fis*-operon appeared to disrupt the expression of the downstream *fis* gene. This was confirmed by LC-MS/MS proteomics analysis on re-constituted mutants (Supplementary Figures S5A,B); therefore, for simplicity, we refer to the *dusB*/*fis* mutations as *fis* mutations. The proteomics data also showed that the “*fis*_fs early_” mutation was the most severe of the tested *fis* mutations and further showed that the mutation in *rpoA* did not change the relative levels of the α protein (Supplementary Figure S5C).

**TABLE 1:** Mutations in *rpoA* and in the *dusB/fis* operon found after whole genome sequencing of evolved *S. enterica* lineages with deleterious synonymous mutations in *rpsT*.

***rpsT* allele**	**Compensatory mutation^a^**	**Designation in figures**
*rpsT*^+^	–	
T36G	*dusB*(G696A [Trp232*]^b^)	*dusB*_Trp232*_^b^
T36G	*dusB*(del pos 928-936 [*fis* RBS]^c^)	*fis*_Δ__RBS_^c^
T36G	*fis*(A282del [Lys94fs]^d^)	*fis*_fs late_^d^
G48A	*rpoA*(C808T [Leu270Phe])	*rpoA*
G48A	*dusB*/*fis*(-10)^e^	*fis*_–10_^e^
G48A	*dusB*(C928A [Ala312Ala, *fis* RBS]^c^)	*fis*_RBS(SNP)_^c^
G48A	*dusB*(del pos 928-936 [*fis* RBS]^c^)	*fis*_Δ__RBS_^c^
A150C	*fis*(C76del [Pro26fs]^f^)	*fis*_fs early_^f^
A150G	*rpoA*(C808T [Leu270Phe])	*rpoA*

^*a*^*dusB* is annotated as *yhdG* in the *S. enterica* genome. ^*b*^The mutation changes Trp232 to a stop (*) codon. ^*c*^*dusB* contains the ribosome binding site of fis (Nafissi et al., 2012). The affected nucleotides in the *dusB* mutants were all located in a predicted stem loop structure, important for ribosome binding and translation of *fis*. ^*d*^The mutation leads to a frameshift five aa from the C-terminal (KYGMN* to NTA*). ^*e*^The mutation changes the putative -10 promoter element for the *dusB/fis* operon from TAATAT to TAATGT. ^*f*^The mutation leads to a frameshift at codon 26 and a premature stop 8 codons later.

### *rpoA* and *fis* Mutations Specifically Compensate for the rpsT Mutations

To examine the compensatory specificity of the *rpoA* and *fis* mutations, we first re-constructed all mutations in wild-type background and in the background of the *rpsT* mutant in which they were originally selected (T36G, G48A, A150C, or A150G; Supplementary Table S3). In addition, the *rpoA* and some of the *fis* mutations were introduced into *rpsT* mutants other than where they were selected to test the specificity of the compensation mechanism. Furthermore, a constructed *fis* deletion mutation was introduced into the wild-type and the four S20 deficient *rpsT* mutants. Growth rates in early exponential phase were measured for all mutants (Figures 2A–C), and fitness in competition experiments was determined in a subset of the strains (Figure 2D). With the exception of the severe *fis* mutations *fis*_fs early_ and Δ*fis*, all tested combinations of *rpoA* and *fis* mutations, in the background of the synonymous mutations, partially or fully restored both exponential growth rates and competitive fitness. For the *fis*_fs early_ and Δ*fis* mutants, the growth rate in exponential phase was independent of the deleterious *rpsT* mutation, increasing the relative growth of mutant A150C from 60 up to 80%, whereas the relative growth rates of the wild-type and the T36G, G48A, and A150G mutants were reduced to 80% (Figure 2C). This finding indicated pleiotropic deleterious effects in *fis* knockout strains, as previously reported (Nilsson et al., 1992). The results agreed with the reduced viable count for the *fis*_fs early_ mutants and the normal viable count in the less severe *fis*_–10_ mutant (Supplementary Figure S6).

**FIGURE 3:**
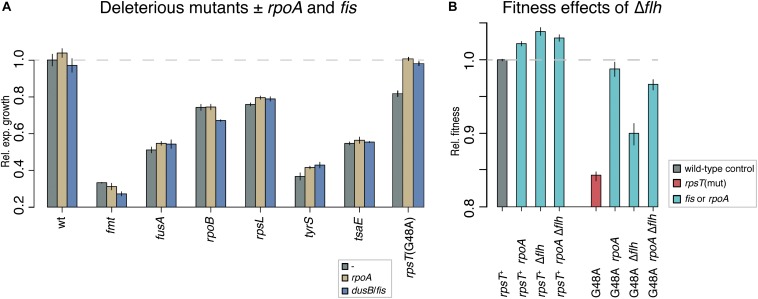
**(A)** Relative exponential growth rate of deleterious mutants with and without the evolved *rpoA* and -10 *dusB*/*fis* mutations. Reported values represent the mean (±SD) of at least three independent experiments. The tested mutations include: an *fmt* mutation (methionyl-tRNA formyltransferase; Thr12Arg) that causes resistance to actinomycin and leads to dysfunction in translation initiation, a *fusA* mutation (elongation factor G; Pro413Leu) causing resistance to fusidic acid and defects in translation elongation and termination, an *rpoB* mutation (RNA polymerase subunit B; Ser531Leu) that results in resistance to rifampicin, an *rpsL* mutation (r-protein S12; Thr129Gly) that yields resistance to streptomycin, a *tyrS* mutation (tyrosyl-tRNA synthetase; Asn204Ser) that causes resistance to mecillinam and a dysfunction in translation elongation, and a *tsaE* mutation (tRNA threonylcarbamoyladenosine (t^6^A37) biosynthesis protein TsaE; Pro413Leu) also causing resistance to mecillinam. The *rpsT* mutation G48A is shown for comparison. **(B)** Relative fitness in competition.

In a wild-type (*rpsT*^+^) background, only the *rpoA* mutation had any beneficial effect (*p* < 0.0001 according to a two-tailed Student’s *t*-test with equal variance), as it increased competitive fitness above wild-type levels (Figure 2D). This general fitness improvement in wild-type background was lower (2%) than the effect observed in the *rpsT* mutants (up to 15%). To further test the specificity of the compensating effect of the *rpoA* and *fis* mutations, we introduced the *rpoA* and a *fis* mutation into strains carrying other deleterious mutations that affected transcription or translation. Relative exponential growth rates were measured and only very minor effects were observed (Figure 3A). These results showed that *rpoA* and *fis* mutations specifically compensated for the deleterious S20 mutations.

Why is the *rpoA*(Leu270Phe) mutation weakly beneficial on its own? A potential explanation for the benefit of the *rpoA* mutation in *rpsT*^+^ strains was found when comparing the proteomics data from the *rpoA* mutants to the other mutants tested. Through a cluster analysis on the combined dataset, we found that the most affected proteins in the *rpoA* mutants were flagellum and chemotaxis proteins (Supplementary Figure S7). As compared with the isogenic wild-type control, the relative levels of these proteins were decreased to about 20% in the *rpoA* mutant, while the *fis* mutations did not affect the levels of these proteins. In *S. enterica* grown in shaking batch culture in LB, loss of flagellar biosynthesis confer a 4% fitness benefit (Figure 3B; Koskiniemi et al., 2012), similarly to the 2% fitness increase measured for the *rpoA* mutation in the *rpsT*^+^ background. Analyses of dry mass yield, viable count, and optical density measurements in stationary phase cultures also showed small increases in the *rpoA* mutant as compared to the isogenic wild-type strain which further indicates the benefit of the mutation (Supplementary Figure S6 and Supplementary Table S4). However, in the S20-deficient *rpsT* (G48A) mutant, as opposed to the *rpsT*^+^ background, the *rpoA* mutation conferred a much higher fitness increase than a Δ*flh* mutation (Figure 3B). No further increases in fitness were detected for the double *rpoA*Δ*flh*, neither in wild-type (*rpsT*^+^), nor in the G48A mutant. Thus, the *rpoA* mutation confers its beneficial effects through at least two different mechanisms: a relatively small, general, fitness enhancing effect (possibly through downregulation of motility-associated genes) and another mechanism that specifically compensates for the fitness reduction caused by S20-deficiency.

### Mutations in *rpoA* and *fis* Restore the Apparent 30S Assembly Defect

**FIGURE 4:**
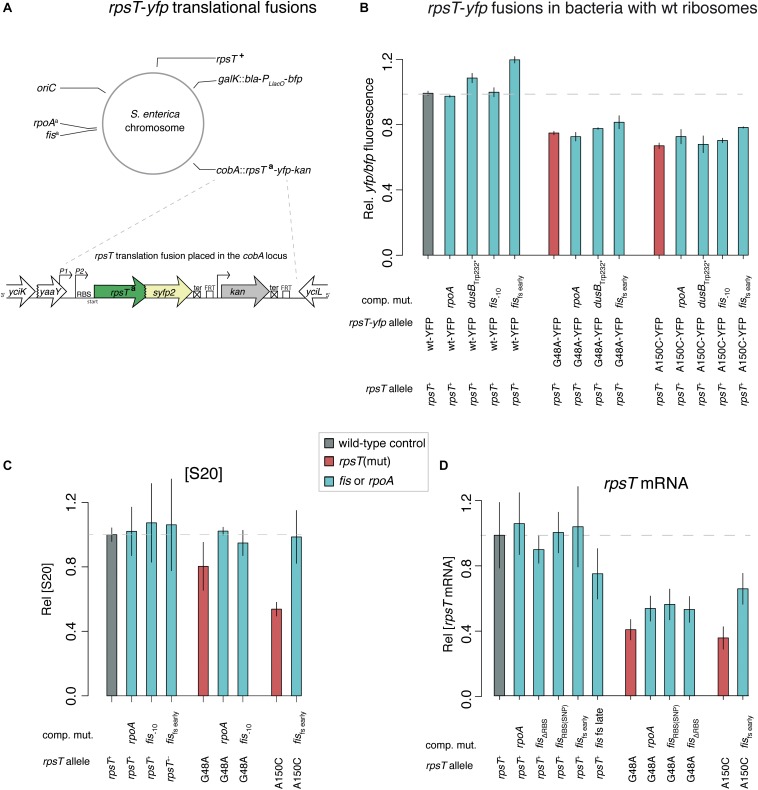
Quantifications of S20 protein and *rpsT* mRNA. **(A)** Schematic illustration of the mutants used in **B**. For all mutants the native *rpsT* locus contain the wild-type allele, allowing the formation of wild-type ribosomes and thus wild-type growth rate (Knöppel et al., 2016). ^*a*^indicates different alleles of the *rpsT*, *rpoA*, and *fis* genes. **(B)** Quantification of S20 through the *rpsT*-*yfp* translational fusions illustrated in A. The *yfp* fluorescence is normalized to *bfp P*_*LlacO*_ fluorescence in the same cells. Reported values represent the mean (±SD) of two independent experiments. See Supplementary Results and Supplementary Figure S4 for measurements in strains with other *rpsT* alleles. **(C)** Quantification of relative [S20] through LC-MS/MS. The values are averages (± SD) of two – four biological replicates normalized to the average of the wild-type from the same 10-plex run. **(D)** Quantification of *rpsT* mRNA through RT-qPCR. Reported values are set relative to an isogenic wild-type control and represent the mean (±SD) of at least five replicates.

**FIGURE 5:**
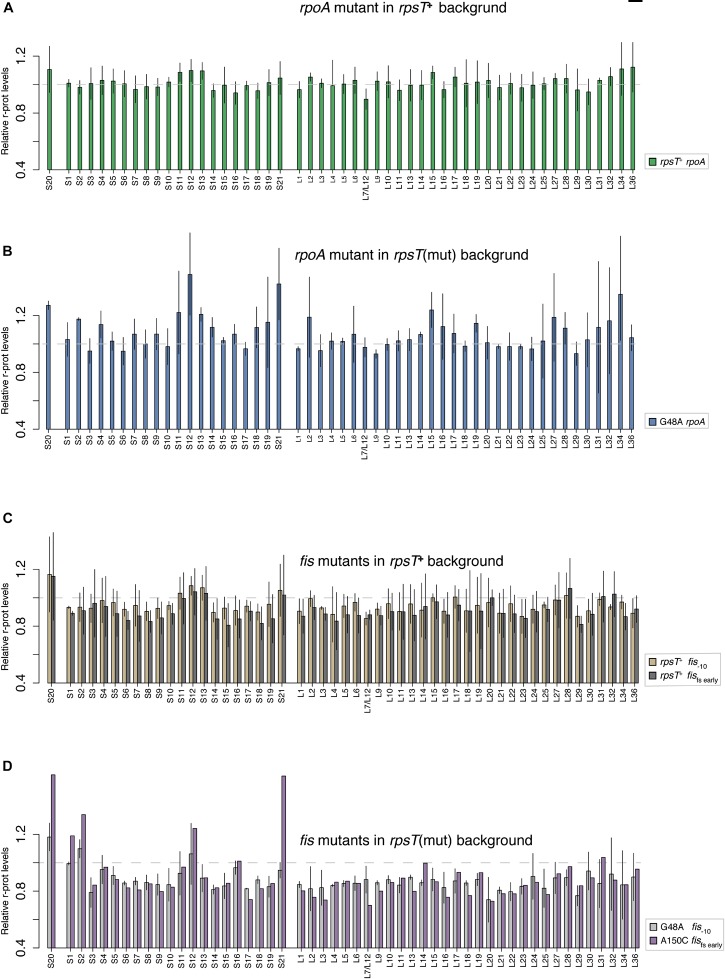
Quantification of relative r-protein levels in *rpoA* and *fis* mutants through LC-MS/MS. **(A)** The *rpoA* mutation in wild-type (*rpsT*^+^) background and **(B)** in the G48A *rpsT* mutant. **(C)** Two different *fis* mutants in wild-type (*rpsT*^+^) background. **(D)** The same two *fis* mutants as in C but in the background of *rpsT* synonymous mutants. The reported values in **A** and **C** are normalized to the average levels of the same protein in the wild-type control (*rpsT*^+^) stain. In panels **B,D**, the values are reported relative to the relevant *rpsT* synonymous mutants (Figure 1A). For values related to the *rpsT*^+^ background please see Supplementary Figure S8. The values represent the average of two or four replicate samples except for the A150C *fis* fs early mutant where only one replicate is presented.

Since *rpoA* and *fis* mutations conferred specific compensating effects on the S20-deficient mutants, we examined if the mutations suppressed the growth phenotype by restoring S20 expression and the 30S assembly defect. To test this, we measured S20 expression in *rpoA* and *fis* mutants using S20-YFP translational fusions and RT-qPCR, and we also analyzed the relative levels of r-proteins by LC-MS/MS (Figures 4, 5). All measurements were performed using exponentially growing cultures. S20-YFP expression was measured in strains having the *rpsT*^+^ allele in its native locus. Hence, the quality of the ribosomes was expected to be normal in the tested strains and not affect the degradation patterns of the *rpsT* mRNA.

For bacteria with fully functional ribosomes (*rpsT*^+^; Figures 4A,B and the first parts of Figures 4C,D, respectively), the results were inconclusive, and, in the majority of cases, no effect on S20 levels was found. If the cause of the benefit of *rpoA* and *fis* mutations was a direct increase of S20, we would have expected a stronger and more consistent positive effect on S20 expression. On the other hand, in S20 deficient bacteria, S20 was clearly increased by the *rpoA* and *fis* mutations (Figure 4C). Similarly, the RT-qPCR measurements revealed that *rpoA* and *fis* mutants showed increased *rpsT* transcript levels only in the S20 deficient strains, which suggests the effect may be secondary (e.g., by increased protection of the *rpsT* transcript due to a higher number of translating ribosomes).

Studying the levels of all r-proteins, it was evident that *fis* mutations increased the four 30S proteins (S1, S2, S12, and S21) to a similar degree as S20 in the S20 deficient mutants, thereby restoring the apparent assembly defect (Figures 1, 5D, and Supplementary Figure S8B). Similarly, but to a lesser degree, the *rpoA* mutations increased the levels of S20, S12, and S21 (Figure 5B and Supplementary Figure S8A). The data from the compensated mutants was normalized in two ways: either to the corresponding *rpsT* single mutant (Figures 5B,D) or to the wild-type (Supplementary Figures S8A,B). Normalizing to the *rpsT* mutant indicates the size of the compensating effect while normalizing to the wild-type instead shows the extent of compensation, i.e., how much closer to wild-type levels the proteins were. With the latter way of normalizing the data, we can see that, as expected, the levels of S20, S1, S2, S12, and S21 appear closer to wild-type levels than they do in the *rpsT* mutants without any compensation. In addition, most of the ribosome associated proteins that were up-regulated in the S20 deficient mutants were restored by the *fis* mutations (in particular RNase P, DeaD, and IF3). However, only a few were restored by *rpoA* mutations (Figure 1B and Supplementary Figure S9).

**FIGURE 6:**
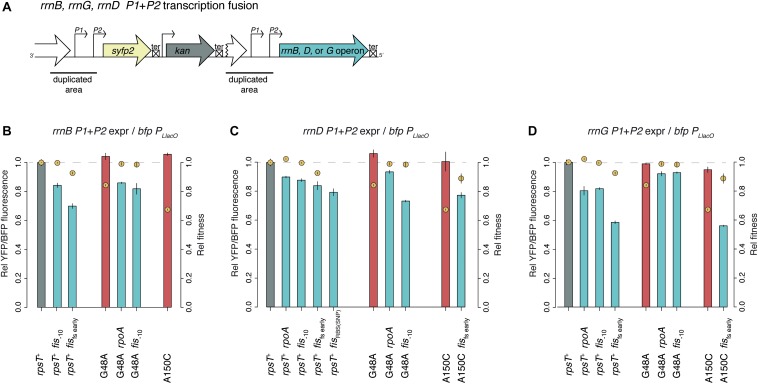
Transcription analysis of *rrn*-operons in the presence and absence of *rpoA* and *fis* mutations. **(A)** Construct of *rrnB*, *rrnD*, and *rrnG* P1 + P2 transcriptional fusions to *yfp.* **(B–D)** Relative *yfp P1* + *P2* fluorescence normalized to *bfp P*_*LlacO*_ fluorescence in the same cells. Relative competitive fitness (±SD) of the same mutants but without the *rrn* constructs is indicated with yellow circles. Expression from *rrnB*, *rrnD*, and *rrnG* was measured in panels **B–D**, respectively. See Supplementary Figures S10, S11 for additional measurements and Supplementary Results for an extended description.

The bulk of r-proteins in the *fis* compensated S20 deficient mutants were downregulated compared to the S20 deficient mutants alone (Figure 5D). Mutations in *fis* even downregulated the r-protein bulk below wild-type levels in both wild-type and *rpsT* synonymous mutant background (Supplementary Figure S8). We suggest that the compensating effect of *rpoA* and *fis* mutations is not via upregulation of S20, as was previously seen for other mutations compensating for S20 deficiency (Knöppel et al., 2016). Instead, we propose that these mutations restored the non-optimal S20:ribosome ratio by downregulating excessive ribosomal components. As synthesis of r-proteins is regulated by the availability of rRNA, the primary reason for the reduced levels of bulk r-proteins could be reduced rRNA transcription. Hence, we measured the expression from the promoter regions of three different *rrn*-operons by *yfp* transcriptional fusions (Figure 6, Supplementary Results, and Supplementary Figures S10, S11). As expected, *rpoA* and *fis* reduced the expression from the three tested promoter regions by between 7–20% (*rpoA*) and 6–42% (*fis*). Surprisingly, despite lower *rrn*-expression, the *rpoA* and *fis*_–10_ mutants maintained wild-type fitness, which is contradictory to the well-known positive correlation between ribosome content and growth rate (Ecker and Schaechter, 1963; Kjeldgaard and Kurland, 1963).

**FIGURE 7:**
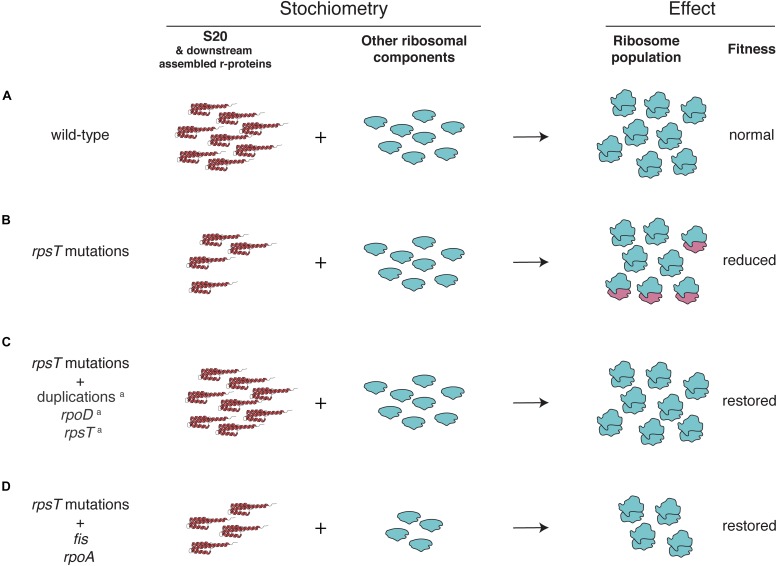
Potential explanations for the fitness reduction observed in the S20 deficient mutants, and fitness restoration by compensating mutations. **(A)** In a wild-type strain production of all ribosomal proteins (including S20 and downstream assembled r-proteins) is balanced to the production of rRNA. **(B)** Synonymous mutations in *rpsT* cause reduced expression of S20 (Knöppel et al., 2016), leading to production of incomplete and dysfunctional ribosomes lacking S20 (Tobin et al., 2010) and downstream assembled r-proteins. **(C,D)** The proportion of functional ribosomes can be restored in two different ways: **(C)** by increased S20 expression and **(D)** by reducing ribosome synthesis. ^*a*^ previously described in Knöppel et al. (2016).

Hypothetically, the skewed stoichiometry between the reduced 30S proteins and the other components of the ribosome in the *rpsT* mutants can be restored in two ways: either through upregulation of S20 (as was seen in our previous study) or by downregulation of other ribosomal components (i.e., ribosomal RNA and other r-proteins; Figure 7). Any deleterious effects of having fewer ribosomes would be outweighed by the improved quality of the 30S pool (i.e., the ribosomes contain S20). The compensatory mutations found in *fis* supports the second scenario. Thus, we see a small and inconsistent increase in the amount of S20 (i.e., effects of *fis* in *rpsT*^+^ background; Figures 4A,B and the first parts of Figures 4C,4D), whereas we see a clear decrease in the amount of other ribosomal components in both *rpsT*^+^ and S20 deficient strains (Figures 5C,D, 6). The effects of the *rpoA* mutation on *rrn*-expression and r-protein levels were smaller than the effects of *fis* mutations and no changes of S20 or other r-proteins were detected in wild-type background (Figure 5A, Supplementary Results, and Supplementary Figures S10, S11). However, in an S20 deficient mutant, *rpoA* mutations increased the levels of S20 and all but one of the other affected 30S proteins (Figure 5B). Although most of the other r-proteins were unaffected, expression decreased from the three tested *rrn*-operons suggesting that this mutation confers both medium adaptation and a specific compensatory effect on the levels of ribosomal components.

### Ribosomes in the *rpoA* and *fis* Mutants Translate at Near Wild-Type Rates

A potential explanation for why *rpoA* and *fis* mutants could grow at wild-type rates even though they appear to produce fewer ribosomes could be that these mutants are able to maintain total translation capacity at wild-type levels, despite having fewer ribosomes. One possibility to achieve this would be if the mutants had a faster translation elongation rate than the wild-type. To test this hypothesis, we measured the *in vivo* rates of translating a LacZ-YFP fusion protein in a wild-type, an *rpoA* mutant, and a *fis* mutant (see section “Materials and Methods”). The elongation rate (in aa/s, average ± standard deviation, *n* = 3) was measured to 17.8 ± 0.06 for the wild-type, 17.0 ± 0.05 for the *rpoA* mutant, and 16.9 ± 0.3 for the *fis* mutant. Although the differences between wild-type and both of the mutants were statistically significant according to a Student’s *t*-test (*p* < 0.05), they were too small and in the wrong direction to explain the observed results. Another potential, but very difficult to test, explanation to account for the maintained growth rate despite reduced ribosome synthesis could be that the *fis* and *rpoA* mutants, in addition to reducing ribosome synthesis, reduces the need for translation capacity by making the global pattern of gene expression better optimized for growing in that particular condition. Thus, the reduction in translation capacity from making fewer ribosomes could be balanced by medium adaptation effects.

## Conclusion

Mutations in the *rpsT* transcript (G48A and A150C) lead to S20 deficiency and reduced levels of four additional r-proteins (S1, S2, S12, and S21), suggesting a defect in the assembly of the small ribosomal subunit. Although further experiments would be needed to provide direct evidence for misassembled particles, our data is consistent with the accumulation of a subpopulation of aberrant pre-30S particles lacking five r-proteins. E.g., we observed an increase in the levels of proteins expressed from leaderless transcripts, which has been described for ribosomes lacking S1 (Moll et al., 2002; Kaberdina et al., 2009). The fitness cost of the *rpsT* mutants could manifest on several different levels, such as global impairments of translation due to a reduction in the fraction of fully functional ribosomes. Another contributing factor could be that r-proteins and RNA that never gets assembled, or end up trapped in inactive ribosomal particles, cause a futile cycle of synthesis and degradation that wastes energy and traps building blocks in useless intermediates. It has previously been shown for both *E. coli* and *S. enterica* that 30S subunits lacking S20 are defective in translation initiation and docking of the two subunits (Götz et al., 1990; Rydén-Aulin et al., 1993; Tobin et al., 2010). In addition, the abnormal 30S subunits can probably block functional ribosomes from binding and initiating translation of the same mRNA (Knöppel et al., 2016). The proteomics analyses indicate that the cells attempt to compensate for this deleterious effect by increasing the levels of proteins involved in ribosome maturation (Figure 1B).

Our results show that mutations that reduce the amount of Fis restore S20 levels in S20-deficient *S*. *enterica*, and that the amino acid substitution Leu270Phe in *rpoA* has both a specific compensating effect on the S20 deficient *rpsT* mutants and a general fitness-increasing effect that is probably due to downregulation of motility-associated genes (Figure 3B and Supplementary Figure S7). Furthermore, our results suggest that the *rpoA* and *fis* mutations reduce rRNA transcription and thereby restore the S20:rRNA stoichiometry and bacterial fitness to normal (Figure 6). These findings combined with our previous study (Knöppel et al., 2016) show how synonymous mutations can have very strong effects on fitness and that there exist a multitude of solutions to restore fitness to wild type levels by mutations that alter protein levels.

Finally, some of the *rpoA* and *fis* mutants had wild-type competitive fitness and grew with wild-type growth rate in exponential phase (Figures 2A,B), although they appeared to produce fewer ribosomes. Hence, there appeared to be a lack of correlation between *rrn*-expression, ribosome levels and competitive fitness (Figure 6B). As the remaining ribosomes in the *rpoA* and *fis* mutants appeared to translate at normal rates, the reason for their normal growth rates remains unexplained.

## Materials and Methods

### Strains and Media

All experiments were performed with *Salmonella enterica* subsp. *enterica* serovar Typhimurium str. LT2 (designated *S. enterica*) and derivatives thereof. Mutations were transferred between strains through generalized transduction with phage P22 HT 105/1 *int-201* (Schmieger, 1972). All growth was done in LB [5 g L^–1^ yeast extract (Oxoid), 10 g L^–1^ Tryptone (Oxoid), 10 g L^–1^ NaCl, and 1 mM NaOH] or LA (LB solidified with 15 g L^–1^ agar). To simplify preparation of competent cells for electroporation, NaCl was omitted from the LB medium. When needed, antibiotics were added to the following concentrations: chloramphenicol (cam); 12.5 mg L^–1^, ampicillin (amp); 50 or 100 mg L^–1^, kanamycin (kan); 50 mg L^–1^, and tetracycline (tet); 7.5 mg L^–1^. To select for loss of the *cat-sacB* cassette, we used sucrose selection plates (LA without NaCl, supplemented with 50 g L^–1^ sucrose). When growing cells to exponential phase to prepare samples for LC-MS/MS, FACS analysis or mRNA extractions for RT-PCR, LB was supplemented with 2 g L^–1^ glucose in order to allow for exponential growth at higher cell density. All growth (except during λ red recombineering) was at 37°C.

### Re-Constitution of Evolved Mutants and Construction of Strains for Competitions

As described in Knöppel et al. (2016). All primers used in the constructions are listed in Supplementary Table S5.

### Construction of Δ*fis*:kan Mutants

The *fis* gene was exchanged by a *kan*^*R*^ resistance marker [amplified from pKD4 (Datsenko and Wanner, 2000)] through λ red recombineering (Datsenko and Wanner, 2000; Yu et al., 2000).

### Transcriptional and Translational Fusions

For the translational fusions of *rpsT* to the *yfp* reporter gene (Figure 4 and Supplementary Figure S4), we moved a constructed fusion of two thirds of *rpsT* to a *yfp* reporter gene [*rpsT*^∗^:*yfp*_[*rpsT*^*xx*^] (Knöppel et al., 2016)] by P22 transductions into the strains with *rpoA* and *fis* mutations and selected for Kan^*R*^. The resultant strains thus had the full-length *rpsT* (wild-type or synonymous mutant alleles) in its native locus whereas the fusion was placed in the *cobA*-locus. For the fusions, we introduced both *rpsT*(wt) alleles and alleles with the synonymous mutations. As an internal reference to compensate for differences in e.g., global translation efficiency that would affect translation from the transcript containing the YFP fusion, we additionally inserted a *bfp*-*amp* cassette (*bfp* expressed under *P*_*LlacO*_) in the *galK* operon. YFP and BFP fluorescence were measured in exponentially growing cells using a MACSQuant VYB Flow Cytometer (Miltenyi Biotec) as described in Knöppel et al. (2016).

For the S6 and S12 *yfp* translational fusions (Supplementary Figures S2A,B) the *yfp* and *kan*^*R*^ were PCR amplified from a strain carrying the *yfp* gene [s*yfp2* (Kremers et al., 2006; Gullberg et al., 2014)] upstream of a *kan*^*R*^ cassette that originates from pKD4 (Datsenko and Wanner, 2000). The amplified fragments were recombined into the reconstructed *rpsT*-mutant strains through λ red recombineering (Datsenko and Wanner, 2000; Yu et al., 2000), producing Duplication-Insertions (Dup-Ins) as described in Näsvall et al. (2017). These Dup-Ins included the S6 or S12 promoters and start codons, the *yfp* cassette fused in frame, and the *kan*^*R*^ cassette followed by the native S6 or S12 operons. Subsequently, the fusions were transduced into re-constructed *rpsT*, *rpoA*, and *fis* mutants. The *bfp*-*amp* internal reference was introduced and fluorescence was measured as described in Knöppel et al. (2016).

In a similar manner, transcriptional fusions to the *rrnB* P1 and *rrnB*, *rrnD* and *rrnG* P1 + P2 promoters (Figure 6A) were constructed and measured. A subset of the *rrn P1* + *P2* strains contained a *bfp*-*amp* cassette (*bfp* expressed under *P*_*LlacO*_) in the *galK* operon.

### Measurement of Dry Weight and CFU Count

A total of thirty cultures of each strain (all originating from one single colony) were grown over night under the same conditions as the evolution experiment (10 ml tubes with 1 ml LB, under agitation at 37°C). The cultures were pooled and OD_600_ was measured using a Shimadzu UV mini 1240 spectrophotometer. For CFU counts, the cultures were serially diluted in PBS and plated on LA plates. To measure the dry weight [according to Mikkola and Kurland (1992)], 25 ml of the overnight cultures were filtered onto Omnipore^TM^ Membrane filter papers. The filters were dried in an oven at 100°C overnight and weighed. The experiment was repeated twice.

### Competitions and Exponential Growth Rate Measurements

Briefly, head-to-head competitions were performed by mixing the mutant strain with the wild-type at 1:1 ratio and, following their relative abundance over time (3 days, 30 generations), each day passaging the cultures 1:1000 in fresh medium. Fluorescent markers (sYFP2 and BFP) introduced into a neutral position of the genome were used to distinguish between the strains. In all competitions, we swapped the markers to compensate for any variation in cost between the two markers. Ratios of YFP and BFP expressing cells were determined using a MACSQuant VYB Flow Cytometer (Miltenyi Biotec). Selection coefficients were calculated using the regression model *s* = {ln[R(*t*)/R(0)]}/*t* (Dykhuizen, 1990), where R is the ratio of mutant to wild-type and *t* is the number of generations. Exponential growth rate measurements were performed by diluting overnight cultures 1:1,000 in fresh media (LB), and thereafter the increase in optical density (OD_600_) over time was measured using a Bioscreen C Reader (Oy Growth Curves) at 37°C with shaking. Growth rates during exponential phase were calculated and normalized to the growth of isogenic wild-types included in each experiment.

### Reverse Transcriptase Quantitative PCR (RT-qPCR)

RNA was prepared from exponentially growing cultures using the RNeasy Mini Kit (Qiagen) and DNase treated using the Turbo DNA-free kit (Ambion). The RNA was then reverse-transcribed into cDNA using the High Capacity Reverse Transcription kit (Applied Biosystems) according to the manufacturer’s instructions. The PerfeCTa SYBR Green SuperMix (Quanta Biosciences, Gaitheerburg, MD) was used for the quantitative PCR reactions according to the manufacturer. The two reference genes *cysG* and *hsaT* were used for normalizations.

### Generation of a Translation Rate Reporter

A *lacZ*-*yfp* translational fusion was constructed by Dup-In recombineering (Näsvall et al., 2017). Two PCR products were generated: (i) containing a 40 bp homology extension toward the end of the *lacZ* gene (excluding the stop codon), a short linker (encoding a flexible gly-gly-gly-gly-ser linker peptide), a partial *syfp2* gene (bps 4–675 of 720), and the *amilCP* and partial *cat* gene (bps 1–606 of 663) from *Acatsac1* (GenBank: MF124798) and (ii) containing the partial *cat* gene (bp 330–663), and *sacB* from *Acatsac1*, a partial *syfp2* gene (bp 31–720) and a 40 bp homology extension toward the intergenic region between *lacZ* and *lacY*. These PCR products were co-transformed into DA59110 (*S. enterica* containing the plasmids pSIM5-Tet and F′128 [*proAB*+ *lac*+]), generating the duplication-insertion *lacZ*-*yfp*’(4–675):*Acatsac1*:’*yfp*(31–720) on the F′ plasmid. The resulting transformants were chloramphenicol resistant (conferred by *cat*), blue (conferred by the blue chromoprotein from *Acropora millepora*, encoded by the *amilCP* gene), and sucrose-sensitive (conferred by *sacB*). In addition, they were slow-growing on minimal medium containing lactose as sole carbon source (probably due to reduced expression of the lactose importer LacY caused by the insertion of the *Acatsac1* cassette which contain transcriptional terminators) and non-fluorescent. During growth on lactose, faster-growing, white, fluorescent clones appeared through homologous recombination between the partial *syfp2* genes, resulting in deletion of the *Acatsac1* cassette and generating the final fusion with a complete *syfp2* gene (*lacZ*-*yfp*). The F′ plasmid containing this fusion was conjugated into recipient strains containing *galK*:*bla*-*P*_*LlacO*_-mTagBFP2 and a deletion of the chromosomal *proAB* operon, selecting ampicillin resistant proline prototrophs (conferred by *bla* in *galK* and *proAB*+ on the F′ plasmid).

### Determination of *in vivo* Translation Elongation Rates

Translation elongation rates were measured by steptime assays, essentially as described previously (Andersson et al., 1982), but using a *lacZ*-*yfp* translational fusion and measuring the accumulation of yellow fluorescent protein (YFP) by flow cytometry instead of measuring β-galactosidase activity. Strains containing a derivative of plasmid F′128 (Kofoid et al., 2003) carrying the *E. coli lac* operon with a *lacZ*-*yfp* fusion were assayed during late exponential growth as expression of *lacZ* was not sufficiently induced earlier in exponential phase. Cultures of three biological replicates of each strain were grown overnight in 1 ml LB, diluted 100-fold in 50 ml fresh medium, and grown to OD_600_ ∼0.65. A 100 μl aliquot was withdrawn into 1 ml phosphate-buffered saline containing 65 mg/L chloramphenicol (PBS + cam; to stop translating ribosomes) at time *t* = 0, after which expression of the *lac* operon was induced by addition of IPTG to a final concentration of 1 mM. For the next 3.5 min, samples were withdrawn to PBS + cam every 10 s. The samples were incubated at room temperature at least 30 min to allow maturation of YFP prior to analysis by flow cytometry using a MACSQuant VYB Flow Cytometer (Miltenyi Biotec). The square root of the background corrected YFP fluorescence intensity (√[*E*(*t*)–*E*(*0*)]) was plotted against time after induction (*t*). The time *t*_*x*_ from addition of IPTG to the appearance of the first complete *lacZ-yfp* fusion peptide was extrapolated from a linear fit to this plot. Assuming the time from addition of IPTG until initiation of translation from the first *lacZ-yfp* mRNAs is negligible compared to the time needed for translation of the fusion peptide, the average translation elongation speed (step time in aa/s) was calculated by dividing *t*_*x*_ with the number of amino acids for the complete LacZ-YFP fusion protein (1267 aa).

### Proteomic Experiments

Overnight cultures were diluted 100-fold in 25 mL LB supplemented with 0.2% glucose. At OD_600_ 0.2–0.25 the cells were pelleted and washed three times in PBS before being frozen for further sample preparation. The Proteomics Core Facility at Sahlgrenska Academy, Gothenburg University, performed the relative quantification of peptides (LC-MS/MS analyses). Briefly, the samples were homogenized using a FastPrep-24 instrument (MP Biomedicals, OH, United States) and digested with trypsin using the filter-aided sample preparation (FASP) method (Wiśniewski et al., 2009). The peptides were labeled using TMT 10-plex isobaric mass tagging reagents (Thermo Scientific, Rockford, United States) and separated using high-pH reversed-phase fractionation (Wang et al., 2011). The fractions were analyzed by nanoLC-MS on the Orbitrap Fusion Tribrid mass spectrometer (Thermo Fisher Scientific, San Jose, United States) interfaced with Easy-nLC 1000 nanoflow liquid chromatography system (Thermo Fisher Scientific, Odense, Denmark). Database searches and quantification of the LC-MS data were performed using Proteome Discoverer version 1.4 (Thermo Fisher Scientific, Waltham, MA, United States) and the *Salmonella typhimurium* strain LT2 (March 2014, 4542 sequences) proteomic database. The detailed description of the experimental procedures, the database search, and the quantitation can be found in the Supplementary Methods.

### Statistical Analysis

Pearson Correlation coefficients for were calculated using the online tool at http://www.socscistatistics.com/pvalues/pearsondistribution.aspx. The PANTHER overrepresentation test (release 20160715) at http://geneontology.org was performed on the 200 proteins with highest average relative expression in the A150C mutant. The GO Biological process complete Annotation Data Set was used with the Bonferroni correction for multiple testing.

## Data Availability Statement

The mass spectrometry proteomics data have been deposited to the ProteomeXchange Consortium via the PRIDE (Vizcaíno et al., 2016) partner repository with the dataset identifier PXD013041.

## Author Contributions

AK constructed most strains and performed most experiments. JN constructed strains and performed translation rate assays. AK and JN analyzed data. All authors planned the work and wrote the manuscript.

## Conflict of Interest

The authors declare that the research was conducted in the absence of any commercial or financial relationships that could be construed as a potential conflict of interest.
