# Supplementary material for: Synonymous Mutations in rpsT Lead to Ribosomal Assembly Defects That Can Be Compensated by Mutations in fis and rpoA
Source: Front Microbiol. 2020 Mar 6;11:340. doi: 10.3389/fmicb.2020.00340 (PMC7069363; doi:10.3389/fmicb.2020.00340)
Supplement: Supplementary file 1 [file Data_Sheet_1.PDF]

Supplementary Material

Supplementary methods, supplementary results and supplementary figures S1 – S11

Synonymous mutations in *rpsT* lead to ribosomal assembly defects that can be compensated by mutations in *fis* and *rpoA*

Anna Knöppel[1,2], Dan I. Andersson[1], and Joakim Näsvall[1,*]

[1]Department of Medical Biochemistry and Microbiology, Uppsala University, Uppsala, Sweden

[2]current address: Department of Cell and Molecular Biology, Uppsala University, Uppsala, Sweden

*** Correspondence:**

Dr. Joakim Näsvall
joakim.nasvall@imbim.uu.se

frontiers

Supplemental Results

Mutations in *rpoA* and *fis* reduce *rrn* expression

One potential mechanism for how the compensatory mutations in *rpoA* and *fis* could compensate the reduction in S20 levels in the synonymous mutants could be that they downregulate transcription of rRNA and thereby restore S20-to-rRNA ratios to normal levels. To test this idea, we constructed transcriptional fusions of *yfp* to the *P1* promoter of the *rrnB* operon, and to the *P1*+*P2* promoters of the *rrnB, rrnD* and *rrnG* operons (Fig. 4 main text and Figs. S10 and S11) and measured how *fis* and *rpoA* mutations affected their activity. Since *fis* activation differ at different *rrn*-operons in *E. coli* (Hirvonen et al., 2001), we measured the effect of *fis* mutations on the expression from all these promoter fusions. Although the magnitude of the effect differed between *rrn* operons, both *fis* and *rpoA* reduced activity from all promoters. Interestingly, the *rpoA* and some of the *fis* mutants restored fitness of the *rpsT* mutants although the *rrn* expression was reduced well below wild-type levels (*e.g.* Fig. 4 main text).

Since we were concerned that other parameters than *rrn* transcription (for example, differences in cell shape, growth rates or translation capacity) could influence the detected fluorescence, the gene encoding the blue fluorescent protein BFP was introduced as an internal control in similarity with (Maeda et al., 2015). The *bfp* gene was expressed from a constitutive promoter (P_{LlacO}) and placed in a neutral position of the genome (the *galK* gene). Fluorescence from both fluorophores was measured simultaneously in a flow cytometer (Fig. 6B – D main text and Fig. S11C – E). The *rpoA* and *fis* mutations reduced the *P1*+*P2*-*yfp* expression in all cases whereas the *bfp* expression in most cases was increased, confirming that the effects of these mutations were specific for the *rrn* promoters. The mutations also increased *bfp* expression even in the wild-type ($rpsT^{+}$) strain, suggesting that the mutations cause a re-distribution of RNA-polymerase from *rrn*-promoters to other promoters in the genome. We further found that the *rpsT* mutants reduced both *bfp* and *yfp* expression evenly, supporting that the low S20 levels causes a general decrease in translation capacity.

Supplemental Methods

Translation rate assays.

Generation of a translation rate reporter. A *lacZ-yfp* translational fusion was constructed by Dup-In recombineering (Näsvall et al., 2017). Two PCR products were generated; one contained a 40 bp homology extension towards the end of the *lacZ* gene (excluding the stop codon), a short linker (encoding a flexible gly-gly-gly-gly-ser linker peptide), a partial *syfp2* gene (bps 4 – 675 of 720), and the *amilCP* and partial *cat* gene (bps 1 – 606 of 663) from *Acatsac1* (GenBank: MF124798); the other contained the partial *cat* gene (bp 330 – 663), and *sacB* from *Acatsac1*, a partial *syfp2* gene (bp 31 – 720) and a 40 bp homology extension towards the intergenic region between *lacZ* and *lacY*. These PCR products were co-transformed into DA59110 (*S. enterica* containing the plasmids pSIM5-Tet and F'128 [*proAB*+ *lac*+]), generating the duplication-insertion *lacZ-yfp*'(4 – 675)::*Acatsac1*::'*yfp*(31 – 720) on the F' plasmid. The resulting transformants were chloramphenicol resistant (conferred by *cat*), blue (conferred by the blue chromoprotein from *Acropora millepora*, encoded by the *amilCP* gene), and sucrose-sensitive (conferred by *sacB*). In addition, they were slow-growing on minimal medium containing lactose as sole carbon source (probably due to reduced expression of the lactose importer LacY caused by the insertion of the *Acatsac1* cassette which contain transcriptional terminators), and non-fluorescent. During growth on lactose, faster growing, white, fluorescent clones appeared through homologous recombination between the partial *syfp2* genes, resulting in deletion of the *Acatsac1* cassette and generating the final fusion with a complete *syfp2* gene (*lacZ-yfp*). The F' plasmid containing this fusion was conjugated into recipient strains containing *galK*::*bla*-P_{LlacO}-mTagBFP2 and a deletion of the chromosomal *proAB* operon, selecting ampicillin resistant proline prototrophs (conferred by *bla* in *galK* and *proAB*+ on the F' plasmid).

Determination of in vivo translation elongation rates. Translation elongation rates were measured by steptime assays, essentially as described previously (Andersson et al., 1982) but using a *lacZ-yfp* translational fusion and measuring the accumulation of yellow fluorescent protein (YFP) by flow cytometry instead of measuring β-galactosidase activity. Strains containing a derivative of plasmid F'128 (Kofoid et al., 2003) containing the *E. coli lac* operon with a *lacZ-yfp* fusion were assayed during late exponential growth, as expression of *lacZ* was not sufficiently induced earlier in exponential phase. Cultures of three biological replicates of each strain were grown overnight in 1 ml LB, diluted 100-fold in 50 ml fresh medium, and grown to OD_{600} ~0.65. A 100 µl aliquot was withdrawn into 1 ml phosphate-buffered saline containing 65 mg/L chloramphenicol (PBS + cam; to stop translating ribosomes) at time $t = 0$, after which expression of the *lac* operon was induced by addition of IPTG to a final concentration of 1 mM. For the next 3.5 min, samples were withdrawn to PBS + cam every 10 s. The samples were incubated at room temperature at least 30 min to allow maturation of YFP prior to analysis by flow cytometry using a MACSQuant VYB Flow Cytometer (Miltenyi Biotec). The square root of the background corrected YFP fluorescence intensity ($\sqrt{[E(t) - E(0)]}$) was plotted against time after induction (t). The time t_x from addition of IPTG to the appearance of the first complete *lacZ-yfp* fusion peptide was extrapolated from a linear fit to this plot. Assuming the time from addition of IPTG until initiation of translation from the first *lacZ-yfp* mRNAs is negligible compared to the time needed for translation of the fusion peptide, the average translation elongation speed (step time in aa/s) was calculated by dividing t_x with the number of amino acids for the complete LacZ-YFP fusion protein (1267 aa).

Mass Spectrometry Analysis.

Cell Lysis. The samples were homogenized in 300 µl lysis buffer (50 mM Triethylammonium bicarbonate (TEAB; Fluka, Sigma Aldrich) and 2% Sodium dodecyl sulfate (SDS)) with 0.1 mm silica beads using FastPrep®-24 instrument (MP Biomedicals, OH, USA). The beads were centrifuged at maximum speed for 15 min, washed with lysis buffer and centrifuged at maximum speed again. The protein extracts were combined and the protein concentrations were determined using Pierce™ BCA Protein Assay (Thermo Scientific, Rockford, USA) and a Benchmark Plus microplate reader (Bio-Rad Laboratories, Hercules, USA) with BSA solutions as standards.

Protein Digestion and Labeling. Aliquots containing 50 µg of each sample were digested with trypsin using the filter-aided sample preparation (FASP) method (Wiśniewski et al., 2009). Briefly, protein samples were reduced with 100 mM dithiothreitol at 60°C for 30 min, transferred on 10 kDa MWCO Nanosep centrifugal filters (Pall Life Sciences, Ann Arbor, USA), washed with 8M urea and alkylated with 10 mM methyl methanethiosulfonate in 50 mM TEAB and 1% sodium deoxycholate. Digestion was performed in 50 mM TEAB, 1% sodium deoxycholate at 37°C in two stages: the samples were incubated with 500 ng of Pierce MS-grade trypsin (Thermo Scientific, Rockford, USA) for 3h, then 500 ng more of trypsin was added and the digestion was continued overnight. Peptides were collected by centrifugation and labeled using TMT 10-plex isobaric mass tagging reagents (Thermo Scientific) according to the manufacturer's instructions. The labeled samples were mixed, and sodium deoxycholate was removed by acidification with 10% TFA. The combined labeled sample was fractionated using the High pH Reversed-Phase Peptide Fractionation Kit (Thermo Scientific) according to the manufacturers protocol. Eight fractions were collected using elution solvents containing 0.1% of triethylamine and 10.0%, 12.25%, 14.5%, 16.75%, 19.0%, 21.25%, 25.0% and 50.0% of acetonitrile. Alternatively, the combined labeled samples were subjected to reversed-phase high pH fractionation with an ÄKTA chromatography system (GE Healthcare Life Sciences, Sweden) using an XBridge C18 3.5 µm, 3.0x150 mm column (Waters Corporation, Milford, USA). In the ÄKTA, 21 fractions were collected over a 32 min gradient from 7% to 40% solvent B (90% acetonitrile, 10% 10 mM ammonium formate in water at pH 10.00) at the flowrate of 0.4 ml/min; solvent A was 10 mM ammonium formate in water at pH 10.00. The fractions from both fractionation methods were dried in a Speedvac and reconstituted in 20 µl of 3% acetonitrile, 0.1% formic acid for analysis.

LC-MS/MS Analysis. Each fraction was analysed using an Orbitrap Fusion Tribrid mass spectrometer (Thermo Fisher Scientific, San Jose, USA) interfaced with an Easy-nLC 1000 nanoflow liquid chromatography system. Peptides were trapped in a C18 trap column (200 µm x 3 cm, particle size 3 µm) and separated in a home-packed C18 analytical column (75 µm x 30 cm, particle size 3 µm) using a 45 min gradient from 5% to 25% solvent B (98% acetonitrile, 0.2% formic acid), followed by a 5 min gradient from 25% to 80% solvent B. Solvent A was 0.2% formic acid. Precursor ion mass spectra were recorded at a resolution of 120,000. The most intense precursor ions were selected ('top speed' setting with a duty cycle of 3s), fragmented using CID at a collision energy setting of 30, and the MS/MS spectra were recorded in an ion trap. Charge states 2 to 7 were selected for fragmentation. The dynamic exclusion was set to 30 s with 10 ppm tolerance. MS3 spectra for reporter ion quantitation were recorded at a resolution of 60,000 with HCD fragmentation at a collision energy of 55 using the synchronous precursor selection of the 5 most abundant MS/MS fragments.

Proteomic Data Analysis. Data analysis was performed using Proteome Discoverer version 1.4 (Thermo Fisher Scientific, Waltham, USA). The protein database for *Salmonella typhimurium* strain LT2 (March 2014, 4542 sequences) was downloaded from Uniprot. Mascot 2.3.2.0 (Matrix Science) was used as search engine with a precursor mass tolerance of 5 ppm

and a fragment mass tolerance of 0.5 Da. No missed cleavages were accepted, mono-oxidation on methionine was set as a variable modification, methylthiolation on cysteine and TMT-6 reagent modification on lysine and peptide N-termini were set as a fixed modification. Percolator was used for the validation of identification results. A target false discovery rate of 1% was used as threshold to filter confident peptide identifications. Reporter ion intensities were quantified in the MS3 spectra using Proteome Discoverer 1.4 at a mass tolerance of 0.003 Da with a reporter absolute intensity threshold of 2000. The resulting ratios were normalized to a median protein value of 1.0 in each sample. The tables containing the protein identities and relative abundances were processed using Perseus 1.5.1.6 software (Tyanova et al., 2016) using the default settings. Briefly, the data was log2-transformed, the missing values were imputed from normal distribution, the resulting matrix was subjected to the principal component analysis and to k-means hierarchical clustering (Euclidean distance, 300 clusters).

Supplemental Figures

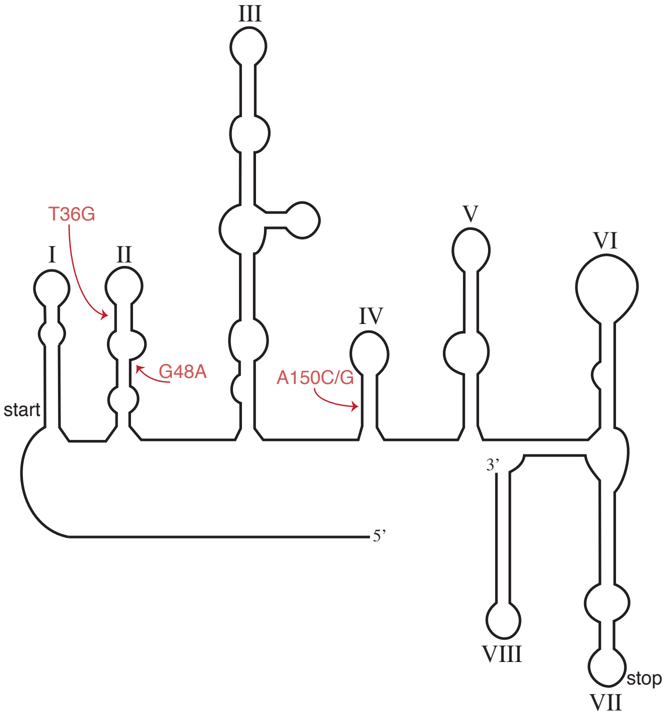

Figure S1. Structure of the *rpsT* P2 transcript (adapted from Knöppel et al., 2016). The four costly synonymous mutations are indicated in red. These include T36G (ValGTT → ValGTG), G48A (LysAAG → LysAAA), A150C (AlaGCA → AlaGCC), and A150G (AlaGCA → AlaGCG). The roman numerals indicate the numbering of the stem-loops in the *E. coli rpsT* transcript (Mackie, 2013).

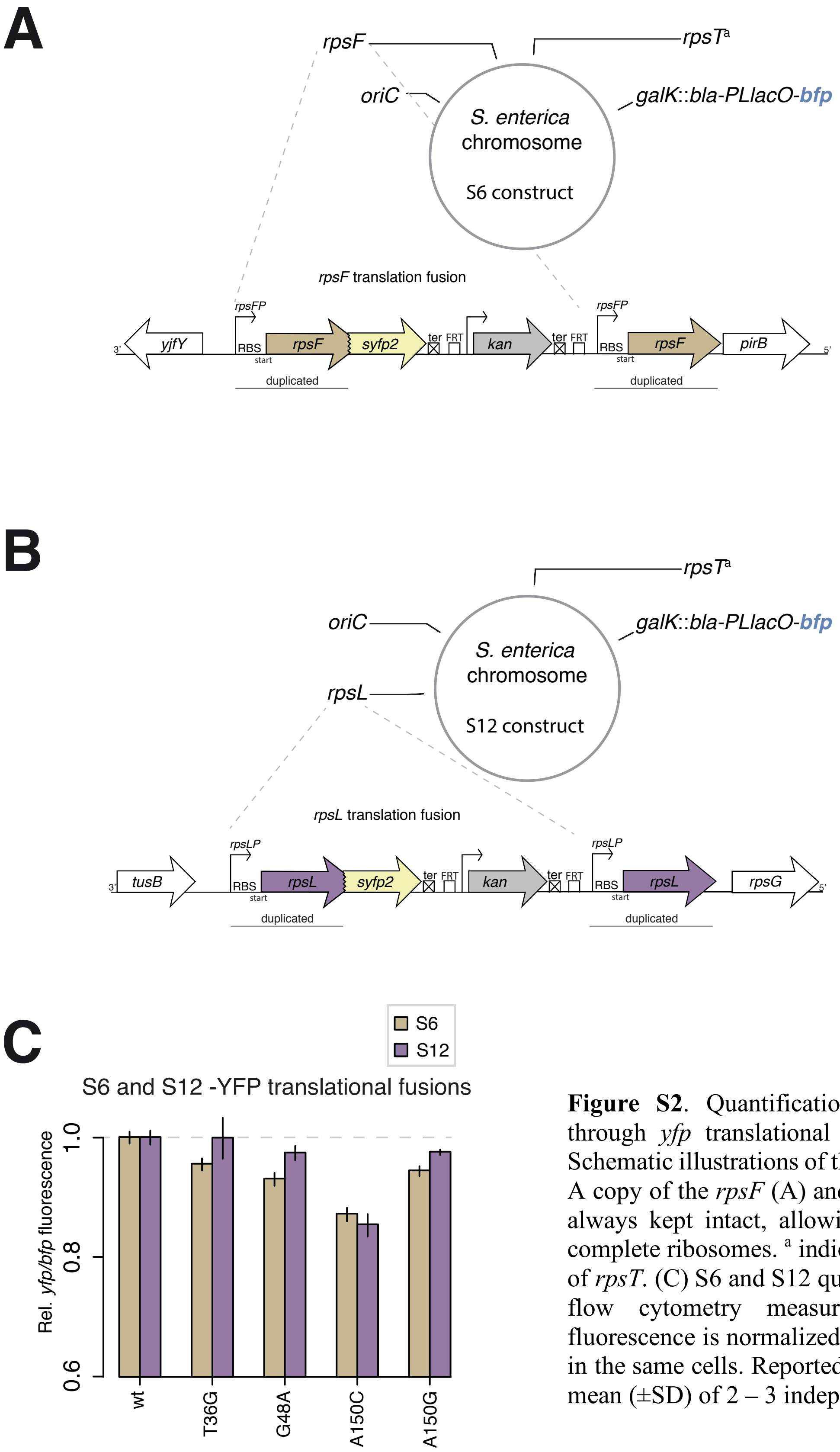

Figure S2. Quantifications of S6 and S12 through *yfp* translational fusions. (A and B) Schematic illustrations of the mutants used in C. A copy of the *rpsF* (A) and *rpsL* (B) allele was always kept intact, allowing the formation of complete ribosomes. [a] indicates different alleles of *rpsT*. (C) S6 and S12 quantifications through flow cytometry measurements. The YFP fluorescence is normalized to BFP fluorescence in the same cells. Reported values represent the mean (±SD) of 2 – 3 independent experiments.

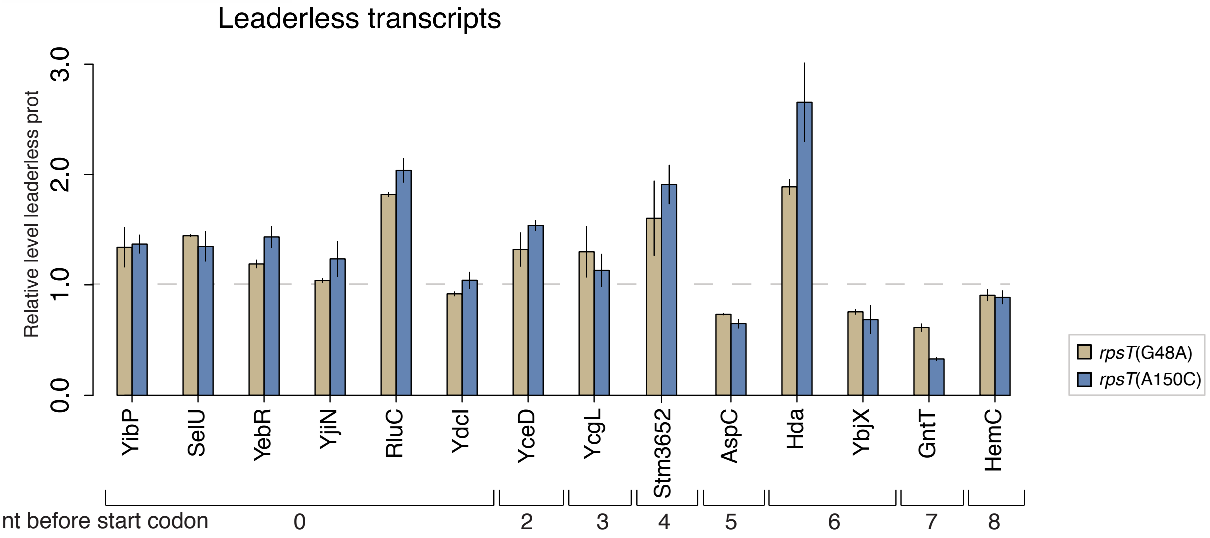

Figure S3. Relative levels of proteins with leaderless mRNAs. The data was obtained through LC-MS/MS and is related to the levels in wild-type.

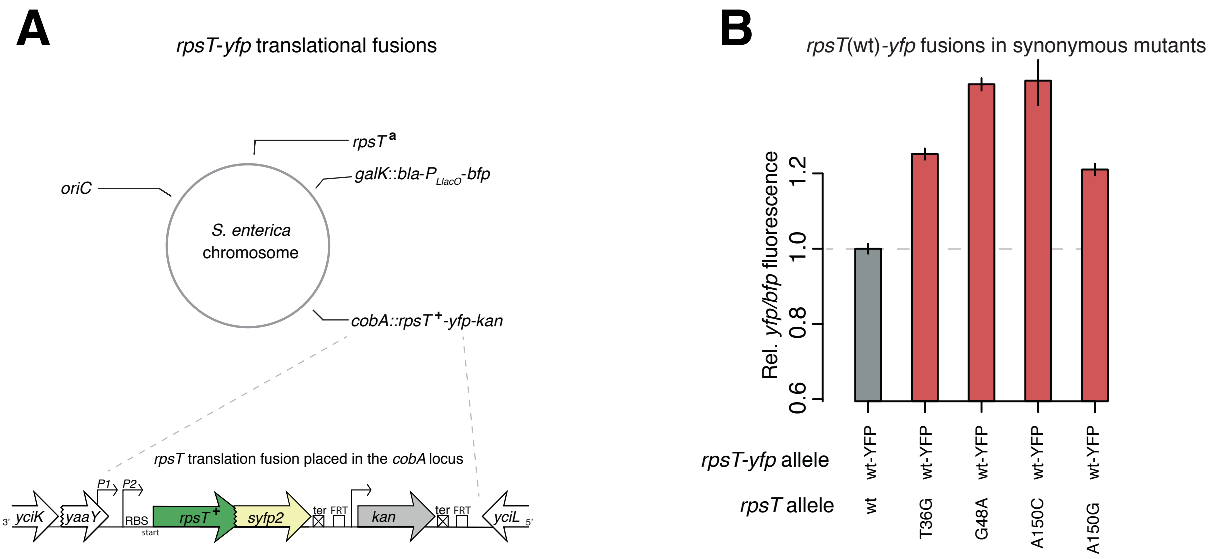

Figure S4. Expression of S20 estimated by *rpsT-yfp* translational fusions. (A) Schematic illustration of the mutants used in B. [a]indicates different alleles of *rpsT*. In all strains, the *yfp* fusion is kept constant (wt *rpsT* sequence) and the allele in the native *rpsT* locus is varied as indicated in B. (B) Quantifications of S20-*yfp* translational fusions. The *yfp* fluorescence is normalized to *bfp* P_{LlacO} fluorescence in the same cells. Reported values represent the mean (±SD) of two independent experiments.

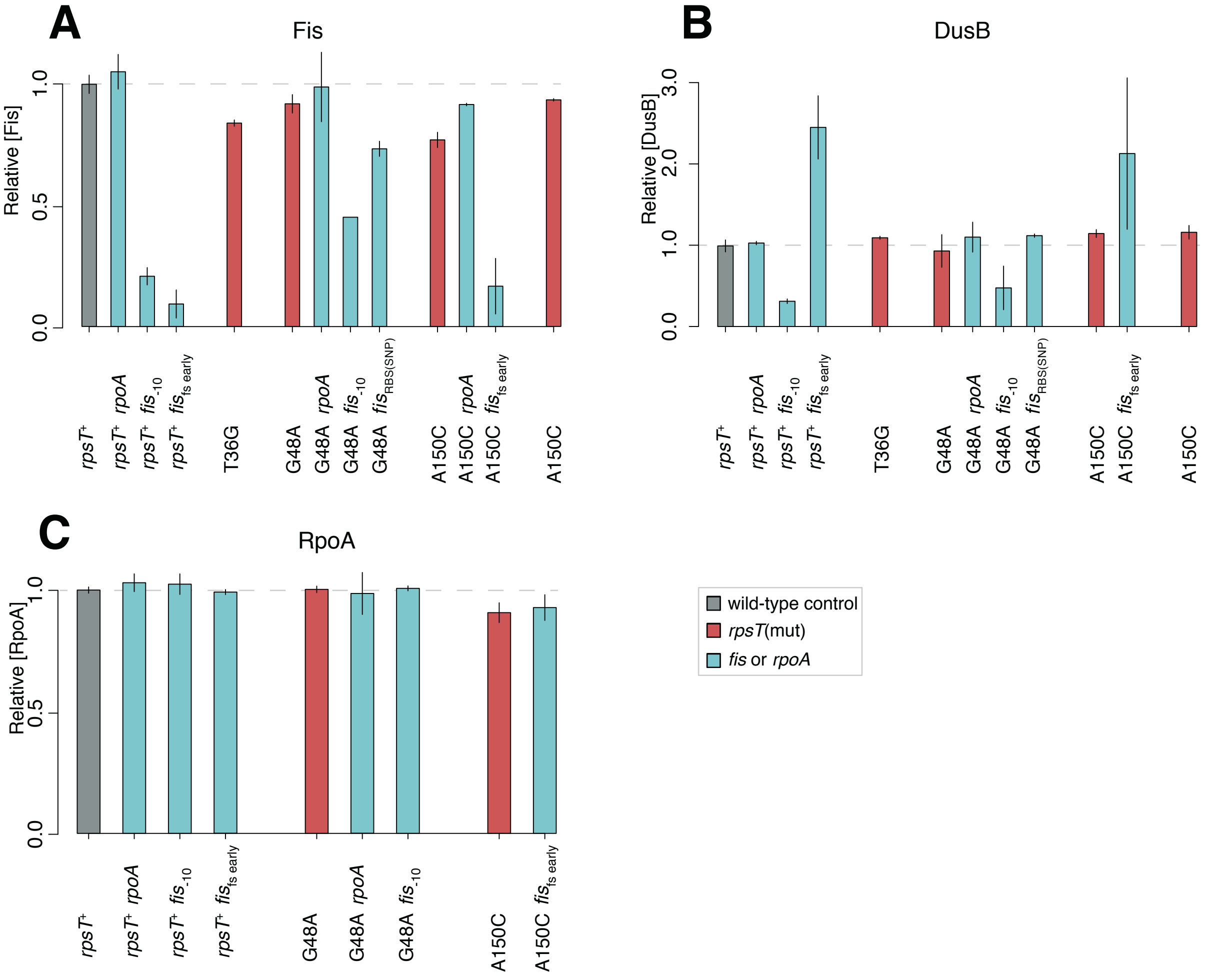

Figure S5. Quantification of Fis, DusB and RpoA through LC-MS/MS. Reported values represents the mean (±SD) set relative to a wild-type control strain. The number of replicate samples varies between 1 and 12 (median 3). (A) Fis. (B) DusB. (C) RpoA. [a] The effect of the *dusB/fis* mutations on tRNA-dihydrouridine synthase B (DusB) levels differed. The promoter mutation downregulated both DusB and Fis. Fis RBS mutation instead up-regulated DusB but downregulated Fis, which is expected since Fis is known to autoregulate the expression of the operon (Ninnemann et al., 1992).

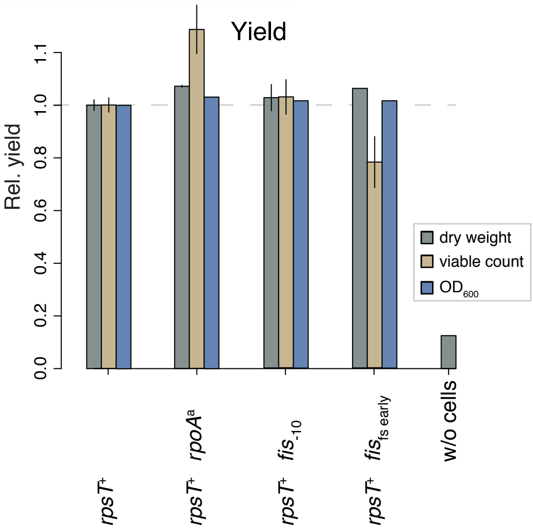

Figure S6. Cell physiology measurements. Relative measurements of dry weight, viable count (CFU), and OD_{600}. See Table S4 for raw data.

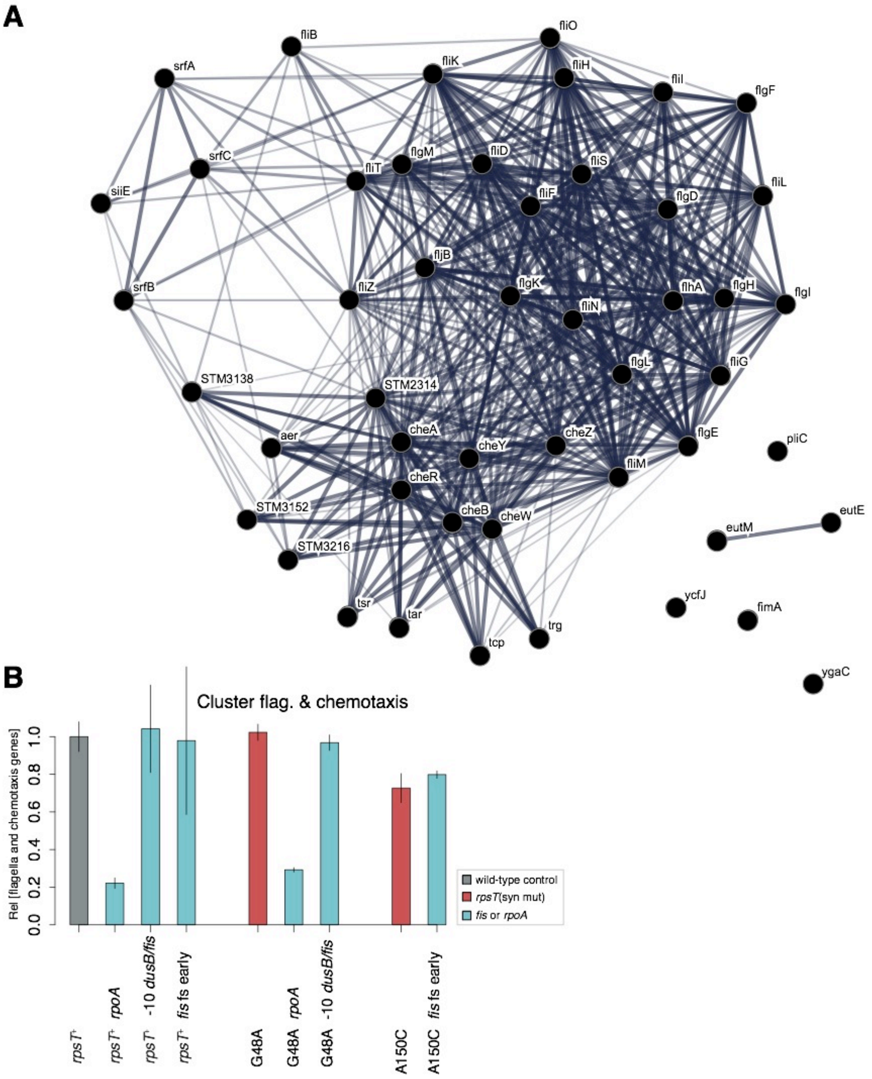

Figure S7. Downregulation of flagella and chemotaxis proteins in *rpoA* mutants. (A) Functional protein association network analysis of the 50 proteins with lowest relative ratio in wt *rpoA* mutants. The large cluster in the middle include flagellar and chemotaxis proteins. PANTHER overrepresentation test (http://geneontology.org (The Gene Ontology Consortium, 2015)) supports the expected overrepresentation of these functional groups among the most under expressed proteins (*p*-values << 0.0001). The cluster analysis was generated using the web-based program STRING version 10.0 (http://string-db.org/ (Szklarczyk et al., 2015)). The thickness of the lines indicates strength of data support. (B) The average of all relative TMT ratios from the clustered flagella and chemotaxis genes in the figure above (40 genes). Error bars indicate the standard deviation between different independent replicates (between 2 – 6).

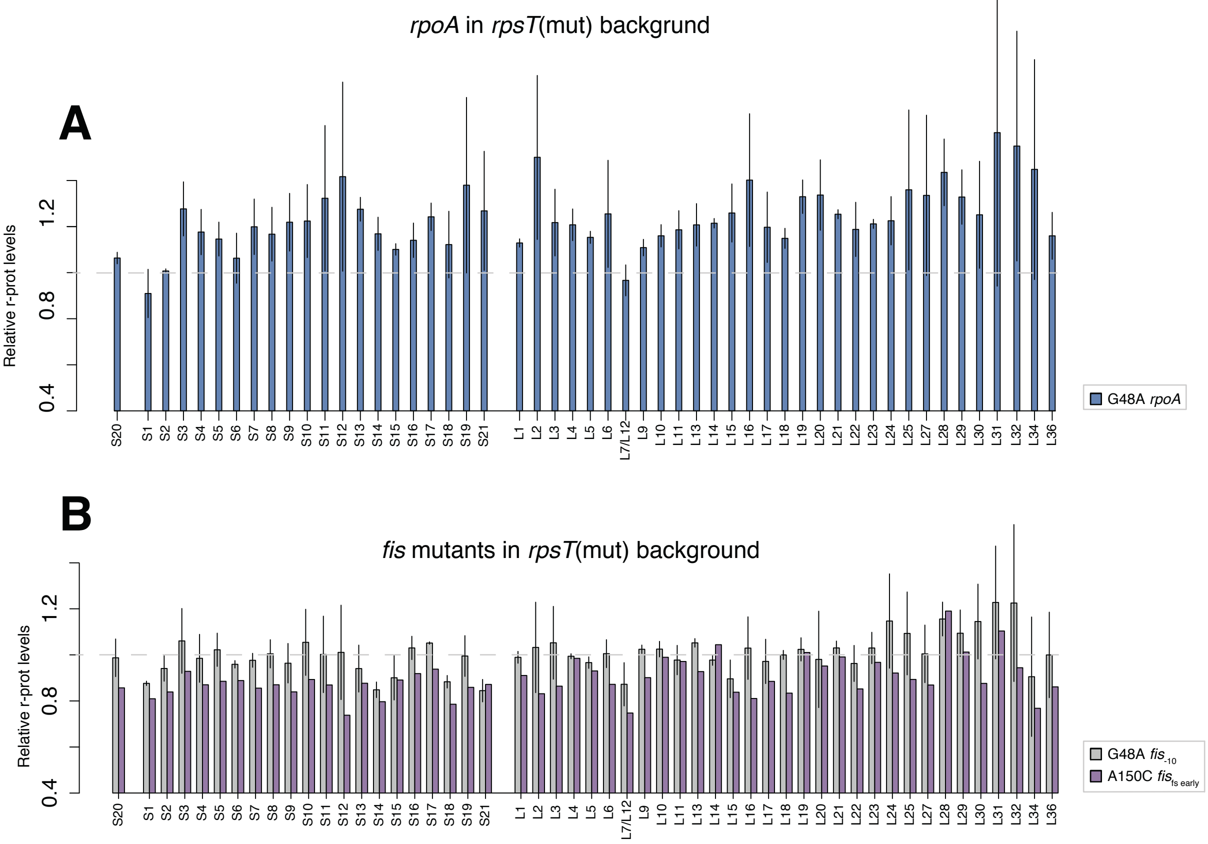

Figure S8. Quantification of relative r-protein levels in *rpoA* and *fis* mutants through LC-MS/MS. The figure shows the same data found in Fig. 5B and D but the values are reported relative to the values for the same proteins in wild-type (*rpsT*$^{+}$) background. (A) The *rpoA* mutation in the G48A *rpsT* mutant. (B) Two different *fis* mutants in the background of *rpsT* synonymous mutants. The values represent the average of two replicate samples except for the A150C *fis* fs early mutant where only one replicate is presented.

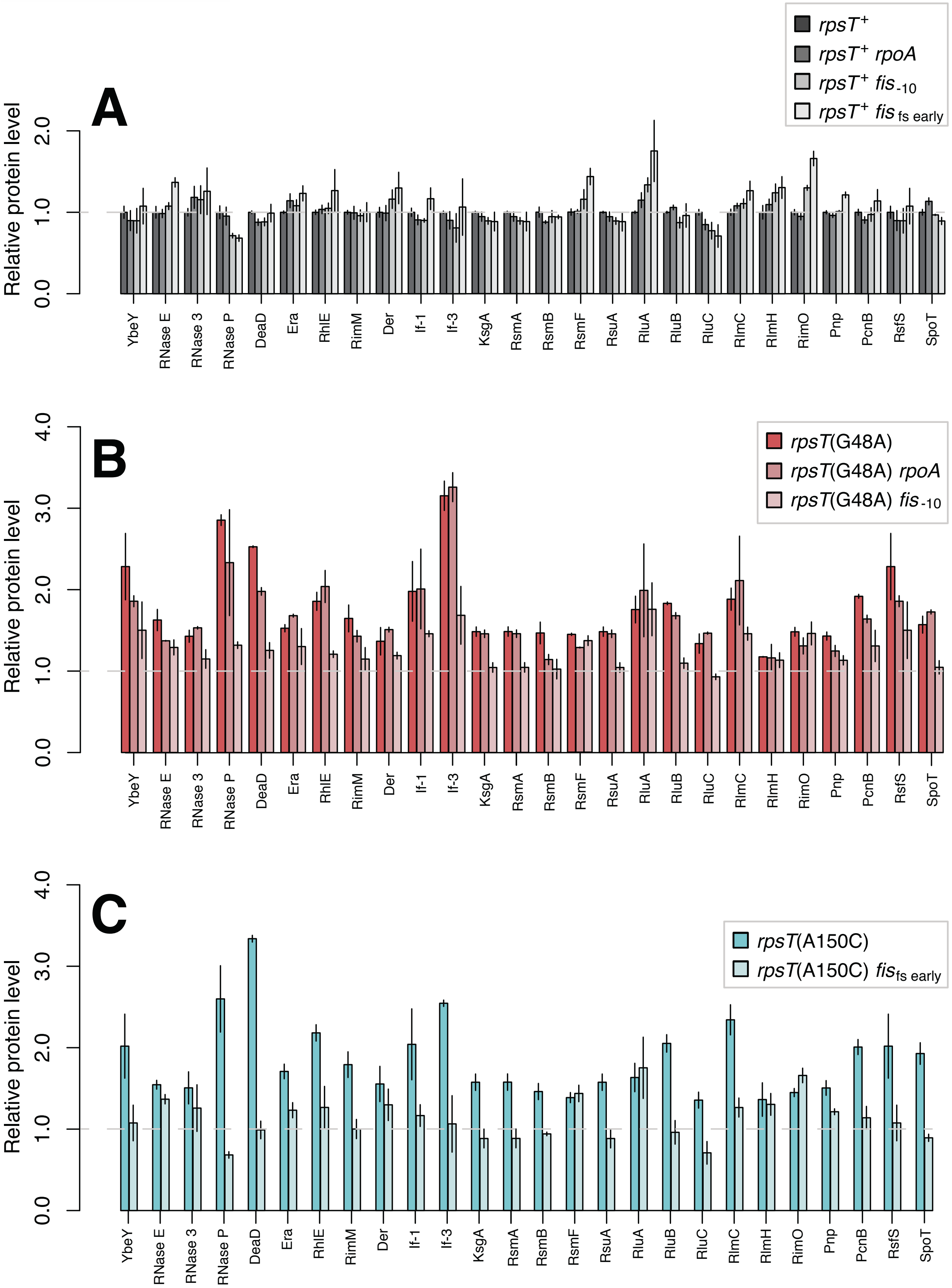

Figure S9. Ribosome associated genes that were found to be upregulated in the S20 deficient mutants. This functional group of genes was overrepresented among the upregulated genes (Table S1).

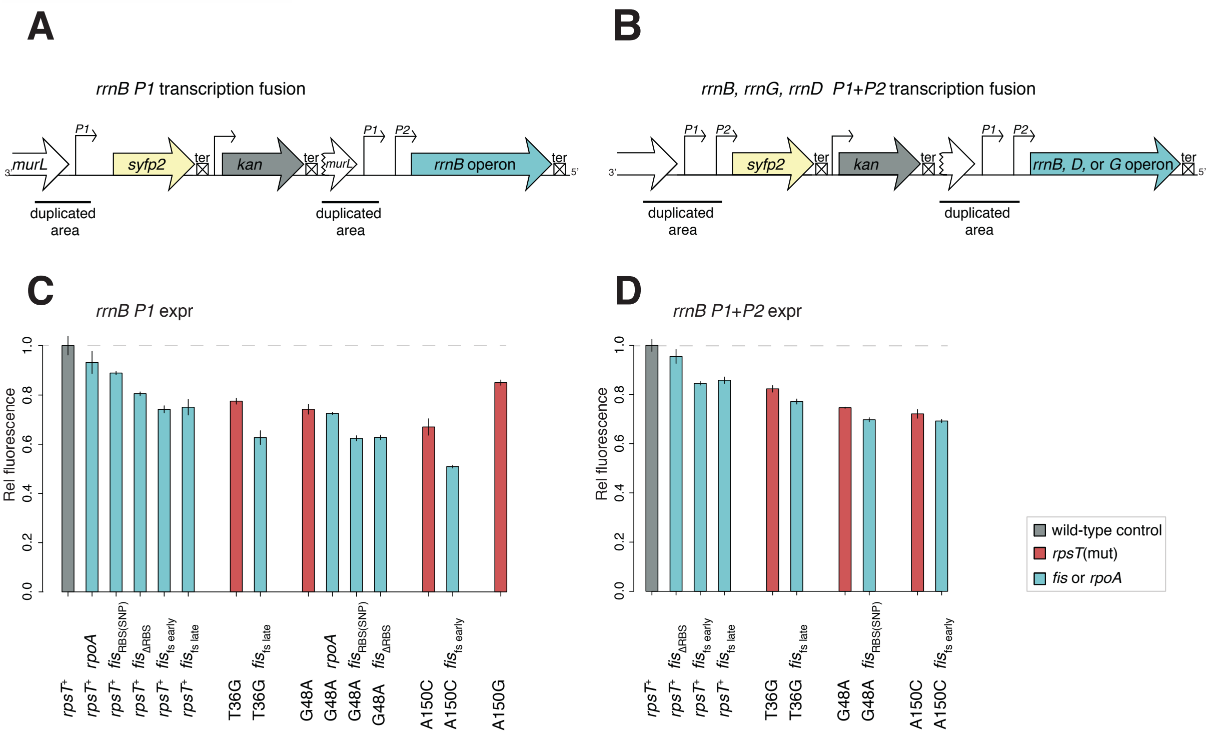

Figure S10. Transcription analysis of *rrn*-operons in the presence and absence of *rpoA* and *fis* mutations. (A) Construct of *rrnB* P1 transcriptional fusions to *yfp*. (B) Construct of *rrnB*, *rrnD* and *rrnG* P1+P2 transcriptional fusions to *yfp*. (C) Relative fluorescence from the fusions in A introduced into a sub-set of *rpsT* mutants with and without *rpoA* or *fis* mutations. Reported values represent the mean (±SD) of four independent experiments made on independently constructed strains. (D) As in C but from the construct in B.

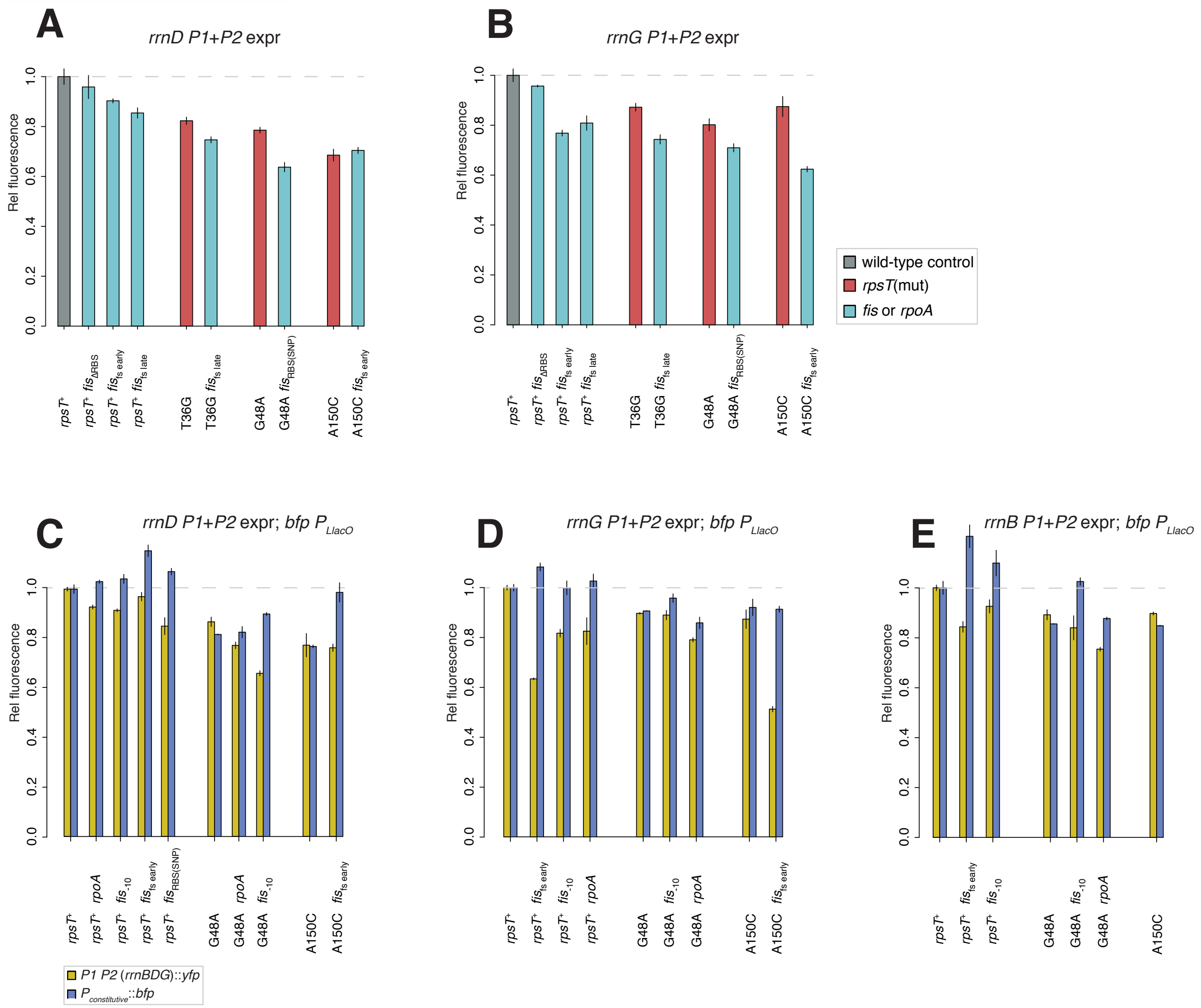

Figure S11. Transcription analysis of *rrn* operons. (A and B) Relative fluorescence from the *rrnD* and *rrnG* fusions in Fig. S10B introduced into a sub-set of *rpsT* mutants with and without *rpoA* or *fis* mutations. (C – E) Relative fluorescence from the *yfp P1+P2* fusions and from *bfp* P_{LlacO} fusions simultaneously measured in the same cells. Reported values represent the mean (±SD) of four independent experiments made on independently constructed strains. Fig. 6B – D in the main text instead pictures the same measurements but the ratio of *yfp* to *bfp* expression.

References

Andersson, D.I., Bohman, K., Isaksson, L.A., and Kurland, C.G. (1982). Translation rates and misreading characteristics of rpsD mutants in Escherichia coli. MGG Mol. Gen. Genet. *187*, 467–472.

Hirvonen, C.A., Ross, W., Wozniak, C.E., Marasco, E., Anthony, J.R., Aiyar, S.E., Newburn, V.H., and Gourse, R.L. (2001). Contributions of UP elements and the transcription factor FIS to expression from the seven rrn P1 promoters in Escherichia coli. J. Bacteriol. *183*, 6305–6314.

Knöppel, A., Näsvall, J., and Andersson, D.I. (2016). Compensating the Fitness Costs of Synonymous Mutations. Mol. Biol. Evol. *33*, 1461–1477.

Kofoid, E., Bergthorsson, U., Slechta, E.S., and Roth, J.R. (2003). Formation of an F′ plasmid

by recombination between imperfectly repeated chromosomal Rep sequences: A closer look at an old friend (F′128 pro lac). J. Bacteriol. *185*, 660–663.
Mackie, G.A. (2013). Determinants in the rpsT mRNAs recognized by the 5′-sensor domain of RNase E. Mol. Microbiol. *89*, 388–402.
Maeda, M., Shimada, T., and Ishihama, A. (2015). Strength and Regulation of Seven rRNA Promoters in Escherichia coli. PLoS ONE *10*.
Näsvall, J., Knöppel, A., and Andersson, D.I. (2017). Duplication-Insertion Recombineering: A fast and scar-free method for efficient transfer of multiple mutations in bacteria. Nucleic Acids Res. *45*.
Ninnemann, O., Koch, C., and Kahmann, R. (1992). The E.coli fis promoter is subject to stringent control and autoregulation. EMBO J. *11*, 1075–1083.
Szklarczyk, D., Franceschini, A., Wyder, S., Forslund, K., Heller, D., Huerta-Cepas, J., Simonovic, M., Roth, A., Santos, A., Tsafou, K.P., et al. (2015). STRING v10: Protein-protein interaction networks, integrated over the tree of life. Nucleic Acids Res. *43*, D447–D452.
The Gene Ontology Consortium (2015). Gene Ontology Consortium: going forward. Nucleic Acids Res. *43*, D1049–D1056.
Tyanova, S., Temu, T., Sinitcyn, P., Carlson, A., Hein, M.Y., Geiger, T., Mann, M., and Cox, J. (2016). The Perseus computational platform for comprehensive analysis of (prote)omics data. Nat. Methods *13*, 731–740.
Wiśniewski, J.R., Zougman, A., Nagaraj, N., and Mann, M. (2009). Universal sample preparation method for proteome analysis. Nat. Methods *6*, 359–362.
